# Y-Chromosome Based Evidence for Pre-Neolithic Origin of the Genetically Homogeneous but Diverse Sardinian Population: Inference for Association Scans

**DOI:** 10.1371/journal.pone.0001430

**Published:** 2008-01-09

**Authors:** Daniela Contu, Laura Morelli, Federico Santoni, Jamie W. Foster, Paolo Francalacci, Francesco Cucca

**Affiliations:** 1 Laboratorio di Immunogenetica, Ospedale Microcitemico, Cagliari, Italy; 2 Dipartimento di Scienze Biomediche, Università di Sassari, Sassari, Italy; 3 Center for Advanced Studies, Research and Development in Sardinia (CRS4), Pula, Italy; 4 Dipartimento di Zoologia e Genetica Evoluzionistica, Università di Sassari, Sassari, Italy; University of Wisconsin, United States of America

## Abstract

The island of Sardinia shows a unique high incidence of several autoimmune diseases with multifactorial inheritance, particularly type 1 diabetes and multiple sclerosis. The prior knowledge of the genetic structure of this population is fundamental to establish the optimal design for association studies in these diseases. Previous work suggested that the Sardinians are a relatively homogenous population, but some reports were contradictory and data were largely based on variants subject to selection. For an unbiased assessment of genetic structure, we studied a combination of neutral Y-chromosome variants, 21 biallelic and 8 short tandem repeats (STRs) in 930 Sardinian males. We found a high degree of interindividual variation but a homogenous distribution of the detected variability in samples from three separate regions of the island. One haplogroup, I-M26, is rare or absent outside Sardinia and is very common (0.37 frequency) throughout the island, consistent with a founder effect. A Bayesian full likelihood analysis (BATWING*)* indicated that the time from the most recent common ancestor (TMRCA) of I-M26, was 21.0 (16.0–25.5) thousand years ago (KYA) and that the population began to expand 14.0 (7.8–22.0) KYA. These results suggest a largely pre-Neolithic settlement of the island with little subsequent gene flow from outside populations. Consequently, Sardinia is an especially attractive venue for case-control genome wide association scans in common multifactorial diseases. Concomitantly, the high degree of interindividual variation in the current population facilitates fine mapping efforts to pinpoint the aetiologic polymorphisms.

## Introduction

Sardinia has long been of interest for human geneticists. Some demographic and genetic features of this population, related to its insularity, offered the opportunity to study the impact of natural selection, for instance in determining resistance against malaria [1] and to clarify relevant aspects of the molecular bases of monogenic diseases common in Sardinia such as Thalassemia [2], Wilson disease [3], and APECED [4]. Now, increasingly, the interest has moved towards common multifactorial diseases. In particular, this island population has, together with Finland, the highest incidence of the autoimmune disease, type 1 diabetes, in the world [5] and represents the main exception to the north-south gradient in disease incidence observed in Europe. Another autoimmune disease, multiple sclerosis, also exhibits a considerably higher incidence in Sardinia compared with all the surrounding Southern-European populations [6]. These data suggest that common type 1 diabetes and multiple sclerosis undetected susceptibility alleles are prevalent in Sardinia and that this population could be a suitable place to look for them using genome wide association scans. However, some key parameters of the genetic structure and past demographic history of this population are still only partially known. How old is this population? What is the explanation for the numerous founder effects observed for the genetic systems so far studied? What was the impact of the various invasions from outside populations on the genetic structure and substructure of the present time population? These issues need to be addressed.

Regarding the age of the population, there is some archaeological evidence of pre-Neolithic occupation after which this island population gradually increased in size during the Neolithic age. An advanced civilisation developed in the Bronze Age, characterised by the building of fortified towers, called *Nuraghe*, throughout the island. During this time (3.6–2.2 KYA) it is estimated that the island population reached approximately 300,000 inhabitants [7] and remained relatively constant during the subsequent Phoenician-Carthaginian (535 BC–238 BC) and Roman dominations (238 BC–476 AD); it did not significantly increase until around 300 years ago. For instance, in 1627 AD a fiscal survey estimated the population to be 297,000. Later, the population was 431,000 in 1781 AD, increased to 636,000 in 1871 AD, exceeded 1,000,000 in 1936 AD and currently totals 1,632,000 individuals.

However, while these archaeological and historical findings provide some interesting clues, they do not give a dynamic portrait of the population history and of the actual impact of these events on the structure of the present-time population. Furthermore, they do not help clarify the early events of the foundation of this population. Genetics can address this in a more informative way.

Previous studies on autosomal variants indicate the absence of significant heterogeneity between large geographic subregions which have suffered, historically, repeated invasions, and the most internal and isolated part of the island which was substantially untouched by these occupations [8], [9]. However, given that this evidence was obtained from autosomal variants, mainly located in the HLA region, whose protein products are under strong natural selection, it is difficult to determine how representative they are for the genome as a whole. Furthermore, some studies have reported a degree of micro-heterogeneity, particularly when comparing small areas and isolated villages [10]–[13].

Hence, interpretation of the overall genetic panorama is not straightforward. Prior knowledge of the genetic structure of a population is relevant not only to reconstruct its ancient demographic history but is also a fundamental prerequisite for association studies, and notably for genome wide association scans to dissect the genetic factors in multifactorial traits. For instance, using the cost effective case-control design in a regionally heterogeneous or substructured population, both false positive and false negative results will occur if patients and controls are not carefully sampled in equivalent proportions according to their origin.

To obtain a more accurate portrait of genetic structure and descent, we determined the Y-chromosome haplotype structure of a large number of Sardinian males using a panel of informative neutral single nucleotide polymorphisms (SNPs) and short tandem repeats (STRs), also known as microsatellites, located in the male specific part of this chromosome. These markers are particularly well suited for these comparative analyses because they were not explicitly affected by evolutionary forces related to the immune response against pathogens. The non-recombining nature of this chromosome region as well as the availability of a reliable molecular clock, through the established mutation rate of STRs, allows a chronological reconstruction of haplotype genealogy, when these different types of markers are analysed jointly [14]–[17]. The aim of the study was thus to establish the intra-regional distribution of these Y-chromosome molecular markers and to infer past peopling and demographic events in Sardinia, evaluating the impact of these factors in association study design for multifactorial diseases.

## Results

**Figure 1:**
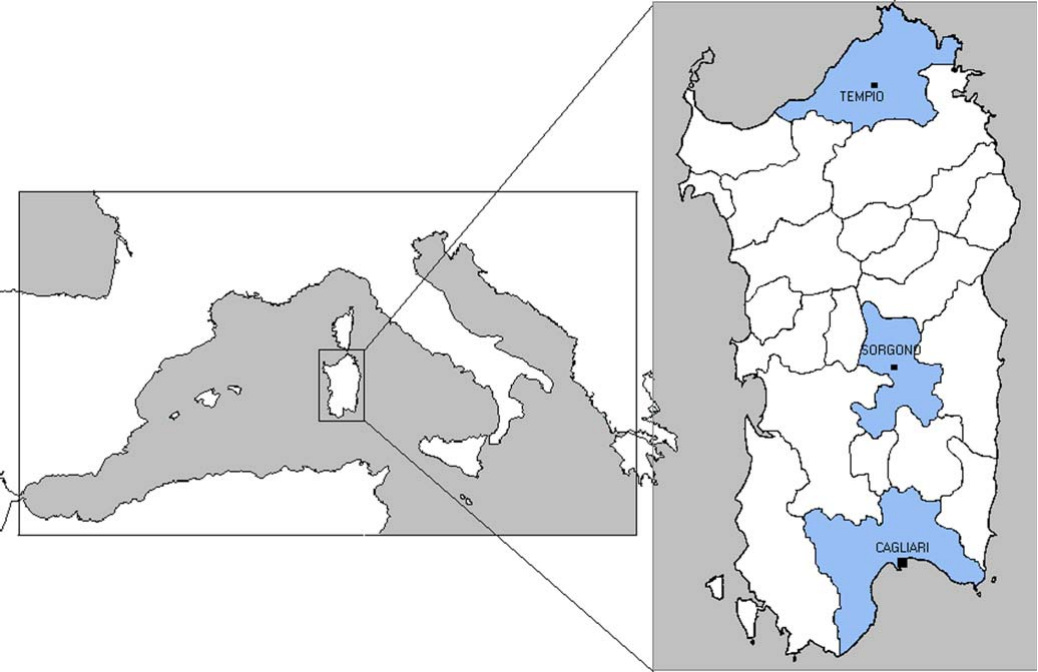
Map of Mediterranean showing the location of Sardinia. Expanded section shows the island of Sardinia with the sample regions highlighted

We established the distribution of the Y-chromosome haplogroup lineages in 376 newborn males from three large regions of Sardinia (Figure 1 and Methods). We found that the distribution of the various haplogroups was relatively homogeneous across these different areas of the island (Table 1). In fact, the pairwise F_STs_ computed from the haplogroup frequencies were not significantly different for inter-population differentiation (Table 2). Similarly, pairwise R_STs_ computed on the same sample using STR markers did not provide significant evidence of inter-population differentiation (Table 2).

**Table 1:** Haplotype frequencies and gene diversity of the Y-chromosome lineages in 376 newborns from three regions of Sardinia

	Cagliari		Sorgono		Tempio	
Haplotype:	N	Frequency	N	Frequency	N	Frequency
M13	1	0.005	0	0.000	0	0.000
M1	1	0.005	0	0.000	0	0.000
M35 (xM78,M123)	1	0.005	1	0.010	1	0.012
M35, M78	12	0.064	3	0.029	3	0.035
M35, M123	4	0.021	1	0.010	4	0.047
M35, ND	3	0.016	3	0.029	3	0.035
M89 (xM170,M172,M201,M9,M267)	0	0.000	0	0.000	3	0.035
M89 (xM170,M172,M201,M9,ND)	2	0.011	1	0.010	0	0.000
M89, M267	5	0.027	3	0.029	0	0.000
M89, M170 (xM26)	6	0.032	0	0.000	0	0.000
M89, M170 M26	59	0.316	38	0.369	24	0.279
M89, M172 (xM102,M92)	5	0.027	0	0.000	2	0.023
M89,M102	5	0.027	3	0.029	0	0.000
M89, M92 (xM67)	3	0.016	0	0.000	1	0.012
M89, M92, M67	7	0.037	3	0.029	3	0.035
M89, ND	2	0.011	0	0.000	1	0.012
M89, M201	26	0.139	12	0.117	18	0.209
M89, M9 (xM173)	2	0.011	2	0.019	6	0.070
M89, M9, M173 (xM17,M18,M269)	4	0.021	0	0.000	0	0.000
M89, M9, M173, M17	1	0.005	3	0.029	0	0.000
M89, M9, M173, M18	3	0.016	5	0.049	0	0.000
M89, M9, M173, M269	33	0.176	22	0.214	17	0.198
M89, M9, M173 (xM17,M18,ND)	2	0.011	3	0.029	0	0.000
Total	187	1	103	1	86	1
Gene diversity (SD)	0.828 (+/−0.019)		0.775 (+/−0.031)		0.821 (+/−0.022)	

ND = not determined, SD = standard deviation

**Table 2:** Population pairwise F_ST_ and R_ST_ values between different Sardinian sub-regions

Pairwise F_ST_ values	Cagliari	Sorgono	Tempio
Cagliari	*	*0.393*±*0.005*	*0.269*±*0.003*
Sorgono	0.000	*	*0.109*±*0.003*
Tempio	0.002	0.009	*

Pairwise R_ST_ values	Cagliari	Sorgono	Tempio
Cagliari	*	*0.310*±*0.005*	*0.898*±*0.003*
Sorgono	0.001	*	*0.212*±*0.004*
Tempio	−0.007	0.005	*

Conventional F_ST_ and R_ST_ values are shown below the diagonal. Corresponding P values with Significance Level = 0.05 are shown in italics above the diagonal.

To further evaluate the impact of population substructure in association studies, we compared the frequencies of biallelic variants from a collection of 399 type 1 diabetes patients, collected from the southern part of the island for a previous study [18] with those from the pool of 376 newborns collected from three geographically distant areas of the island (data referring to the STRs and SNPs fully and jointly typed in the Sardinian samples are reported in the supplementary Table S1). Even under this forced scenario – very different from the preferred collection of patients and controls in similar proportions from the same subregions- we did not obtain any significant evidence of heterogeneity (F_ST_ = 0.001, P = 0.157).

With the information that the Sardinian population was relatively genetically homogeneous, we established the haplogroup frequencies in a joint sample set of the 376 newborns, 399 type 1 diabetes patients, and an additional 155 blood donors. In this enlarged data set of 930 males, we detected 19 haplogroup lineages (Table 3). Haplogroups defined by the M130 and M68 SNPs were tested for, but not detected. We then compared the haplogroup frequencies observed in these 930 Sardinian males with those reported in other Euro-Mediterranean populations. Distribution of the Y-chromosome haplogroups detected in the Sardinians set them apart from the rest of the European groups [14], [16], [19], [20].

**Table 3:** Haplotype frequencies of the Y-chromosome lineages and gene diversity in 930 Sardinian males

Haplotype:	N	Frequency
M13	1	0.001
M1	5	0.005
M35 (xM78,M123)	9	0.010
M35, M78	41	0.044
M35, M123	19	0.020
M35, ND	9	0.010
M89 (xM170,M172,M201,M9,M267)	12	0.013
M89 (xM170,M172,M201,M9,ND)	4	0.004
M89, M267	21	0.023
M89, M170 (xM26)	23	0.025
M89, M170 M26	344	0.370
M89, M172 (xM102,M92)	27	0.029
M89,M102	22	0.024
M89, M92 (xM67)	16	0.017
M89, M92, M67	26	0.028
M89, ND	3	0.003
M89, M201	117	0.126
M89, M9 (xM173)	22	0.024
M89, M9, M173 (xM17,M18,M269)	13	0.014
M89, M9, M173, M17	13	0.014
M89, M9, M173, M18	16	0.017
M89, M9, M173, M269	158	0.170
M89, M9, 173 (xM17,M18,ND)	9	0.010
Total	930	1
Gene diversity (SD)	0.801 (+/−0.010)	

ND = not determined, SD = standard deviation

**Figure 2:**
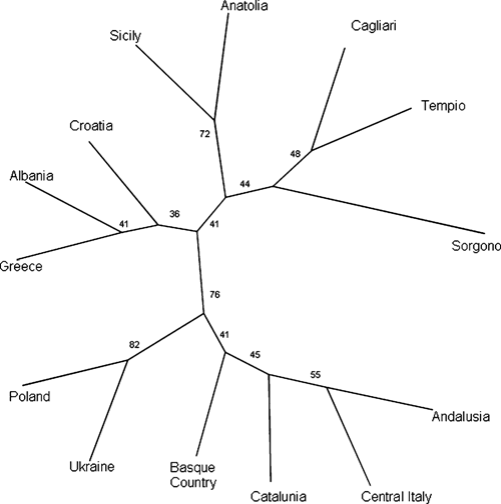
UPGMA consensus tree correlating European populations constructed using 1000 F_ST_ pairwise distances. Numbers before forks indicate percentage of times that each group appeared in the input trees

The main discriminator of Sardinians from the other populations comes from the I-M26 haplogroup. This variant shows an overall frequency of 0.37 in Sardinia but is absent in most other populations, including the neighbouring island of Corsica [19]. It has been detected, though at much lower frequencies, only in the Basques [14], [16], [19], [20] and in a few other western European populations [21]–[23]. In addition, the rare M18 SNP appears to be unique to Sardinia. Furthermore, some haplogroups present and relatively frequent on the island, such as G-M201 and E-M78, have only been detected in eastern countries while another haplogroup common in Sardinia, R-269, is more common in western populations [14]. As a result of this distribution of lineages in Sardinia, there is a high degree of gene diversity (0.801±0.010) (see Table 3) and significant evidence of differentiation from all the other groups for which matched genetic data are available (F_ST_ values and their P values are shown in Tables S2 and S3). An UPGMA (Unweighted Pair Group Method with Arithmetic mean) tree constructed using one thousand bootstrap FST distances, graphically illustrates the differentiation of Sardinians from the other populations (Figure 2).

**Figure 3:**
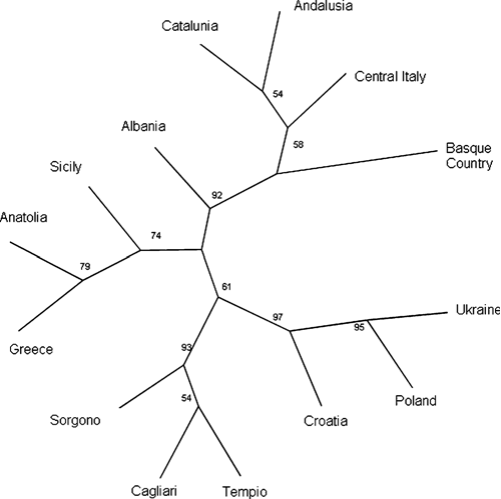
UPGMA consensus tree correlating European populations constructed using 1000 R_ST_ pairwise distances. Numbers before forks indicate percentage of times that each group appeared in the input trees

A possible reservation to this scenario is an SNP ascertainment bias, given that the samples originally screened for Y chromosome SNPs [15] included Sardinian Y chromosomes, and in fact both M18 and M26 were discovered in Sardinian DNA samples. In principle, this kind of ascertainment bias would artifactually increase the differentiation of Sardinians from other populations. However, comparable results were also obtained computing pairwise R_ST_ values (Tables S4 and S5) based exclusively on STR data which are not prone to this bias (see Figure 3 which shows a consensus tree from one thousand bootstraps). We next used BATWING analysis to establish the individual TMRCA for the most representative haplogroup lineages (I-M26, R-M269, G-M201, E-M78), collectively accounting for 71% of the Sardinian Y-chromosome haplogroups, analysed together with the 6 STR markers. The TMRCA values for these haplogroups are included in a span of 19.5–22.8 KYA (Table 4). We also assessed the TMRCA for the rare Sardinia-specific R-M18 haplogroup, and obtained a value of 8.1 (7.8–10.5) KYA.

**Table 4:** TMRCA values of the main Sardinian haplogroups provided by BATWING analysis

Lineage	TMRCA	95% c.i.
I-M26	21.0	16.0–25.5
R-M269	22.8	17.0–25.4
G-M201	19.5	14.0–22.2
E-M78	21.0	19.4–30.8
R-M18	8.1	7.8–10.5

Time is expressed in KYA (thousand years ago)

TMRCA = Time from the most common ancestor

We also used BATWING to analyse the data from the 376 Sardinian newborns to evaluate when this island population began to expand, and obtained a value of 14.0 (7.8–22.0) KYA. To assess this data in the context of that observed in other European populations, we used matched published data [16], [20], [24] and computed the time of initial expansion of other populations of interest. We found a value of 9.5 (6.7–13.7) KYA for the Anatolian population, whereas two data sets of West-Balkan and Iberian populations provided a signal of initial expansion of 7.4 (6.5–16.1) and 9.8 (9.5–13.7) KYA respectively. BATWING analysis also suggested an effective population size at the beginning of the expansion of 789 (450–1264) for the Sardinians, 1741 (957–2857) for the Anatolians, 601 (218–867) for the Balkans and 339 (273–668) for the Iberians.

## Discussion

Our findings indicate that three large, geographically distinct subregions of Sardinia show no significant evidence of inter-region genetic heterogeneity by any of the established measures of population differentiation. In particular, sub-populations from the Sardinian coastal regions (the Campidano and Gallura areas), which suffered cultural and political dominations over many years do not significantly differ from the most internal and isolated part of the island (Barbagia area), which was never under foreign control. This is in agreement with other studies that analysed different chromosomes and independent samples [8], [9].

The homogenous distribution of variants in different macro-areas of the island is consistent with a common ancient origin and cultural background. For example, *Nuraghe*, the characteristic Sardinian prehistoric towers and castles, are widely and evenly distributed across the island and are substantially confined to Sardinia, thus supporting their local creation. At first glance their homogeneous intra-regional distribution could also be explained by mimicry by rival tribes, due to their strategic value. However, the full correspondence of their complex architectural features as well as the overlapping structure of the prehistoric villages surrounding them and the identity of the tools, sculptures, and artefacts of symbolic nature found with them is overwhelmingly suggestive of a common cultural background which may be consistent with internal gene flow and may have acted as a main homogenising force during the Bronze Age.

Our data also provide some clues about the time when the Sardinian population was initially founded. We found that the common Y-chromosome haplogroups detected in Sardinia show rather even TMRCAs ranging from 19.5 to 22.8 KYA, which appear independent from their genealogy and thus indicative of earlier pre-Neolithic founder effects. In particular, the I-M26 haplogroup, showing a TMRCA of 21.0 (16.0–25.5) KYA, is especially informative because it is present in 37% of Sardinian males but is absent or very rare in all other human groups. Furthermore, I-M26 represents a terminal branch of the Y-chromosome tree and is thus a relatively recent haplogroup. Overall, it is therefore conceivable that little intra-haplogroup variability entered for I-M26 at the foundation of the Sardinian population and its TMRCA value is, in fact, roughly related to the initial occupation of the island.

BATWING analysis also reveals that the Sardinian male population began to expand about 14 KYA, with an initial effective population size on the order of 1,000 individuals. The signal of expansion detected by BATWING probably does not predate the arrival of the Sardinian founders on the island, because we observed that other European sample sets provide more recent times of initial expansion.

Thus, despite the simplified demographic model and wide confidence intervals of the BATWING estimates, a coherent picture shows expansion starting in pre-Neolithic times. In fact, the inferred BATWING values are conservative, using a generation time of 25 years. Furthermore, to obtain more robust and reliable results we used mode-based estimates instead of the more commonly-used mean and median measures that tend to provide much older values for all the parameters assessed with BATWING (Methods and unpublished results).

These genetic estimates are in agreement with archaeological evidence suggesting that Sardinia was already populated 18–10 KYA [25], [26]. Furthermore, the genetic data are also consistent with geological data pointing to a land connection between Sardinia and the Italian peninsula (through Corsica) during the last glacial period, which might have allowed the passage of people for several thousand years until about 10 KYA. Evidence of an early pre-Neolithic population has also been derived from other genetic markers, including mitochondrial haplogroups V and H3 [27], [28]. Sardinia may have attracted Palaeolithic hunter-gatherers, with its temperate climate; *Megaceros cazioti* deer and endemic species with a high reproductive rate; and other food sources such as fruits from the forests that covered the island.

The absence of the I-M26 lineage on the nearby island of Corsica [19] is somewhat surprising because Corsica should have been accessible to ancestral Sardinian settlers via a land bridge. However, Corsica may have been resettled from the Italian mainland during medieval times, a hypothesis supported by historical sources. Repopulation could have substituted for I-M26 and masked the putative Corsican ancestral genetic substratum.

The data and conclusions presented in this study, and notably the absence of significant population sub-structure in Sardinia, have a significant impact on the design of genome wide association studies. Robust and statistically well-powered whole genome case-control association studies are thus promising in this population.

Other founder populations, such as the Finnish and the Icelandic, show some substructure in geographically distinct areas [29], [30]. There are indications of micro-heterogeneity in subisolated in Sardinia as well, but they appear to be second-order effects. For example, one survey suggested the presence of heterogeneity among 21 subregions [12]. However, an analysis of molecular variance (AMOVA) using the allele frequencies reported by the authors indicates that more than 99.8% of variation is within the tested subpopulations and less than 0.2% is between them (Table S6).

Another study found some degree of heterogeneity in the distribution of Y chromosome markers of individuals with monophyletic surnames from different sub-regions [10] but, consistent with our results, found no evidence of significant heterogeneity when considering only the place of birth of the genotyped individuals. By selecting solely the monophyletic surnames, a relevant and shared part of the variability is removed from the analysis. Selection of monophyletic surnames is also more prone to the cumulative effect of mispaternity.

Weak evidence of heterogeneity in the distribution of Y chromosome markers from different Sardinian sub-regions was also reported by Scozzari and colleagues [11], but only for one of 17 markers tested, and comparing samples as small as 18 individuals (average of N = 33) and including small towns. Considerable evidence infers many demographic bottlenecks during the settlement of small and dispersed villages present on the island [13], [31], [32] which could account for the micro-heterogeneity and higher degree of background linkage disequilibrium observed in these small and isolated areas compared to the general Sardinian population [13], [31], [32]. Thus, the overall important feature that is confirmed by this study is the lack of significant differentiation between macro Sardinian areas. This finding is consistent with the fact that disorders such as type 1 diabetes and multiple sclerosis exhibit no significant differences in incidence in different Sardinian subregions.

In addition, while we observed a relatively homogenous distribution of genetic markers across Sardinia, we found no restriction of inter-individual variability. For instance, the Sardinians exhibit a higher degree of inter-individual variation than the Spanish population [16], [33]. In general, Y chromosome variants common in either eastern or western European populations are detected at appreciable frequencies in this Mediterranean island. This ensures a high chance to detect, in this island, common ancient disease variants present in both western and eastern Europe that predate the foundation of Sardinia and its separation from the other European populations. Furthermore, these features, together with the fact that the population is ancient, account for highly informative haplotype splits observed in autosomal gene regions across the genome [34]–[36]. This is especially useful during the post-detection fine mapping phase to highlight primarily associated variants in diseases common in this population when it is essential to exclude secondary hitchhiking effects.

The Y chromosome data also clearly underline the uniqueness of the Sardinian population and indicate that strong founder effects are not restricted to mutations involved in monogenic traits such as Beta-thalassemia [2] or Wilson disease [3] but also include neutral, common SNPs, as illustrated by the I-M26 variant. This suggests that by virtue of these founder effects, some disease variants could be unique to the Sardinians or could be in the optimal range of frequency to allow their detection in this population.

Overall these data indicate that Sardinia provides a promising venue for effective genome wide association scans and for the subsequent fine mapping efforts to pinpoint aetiological polymorphisms in multifactorial trait research.

## Materials and Methods

### Sample selection

DNA samples from newborns was extracted using the Chelex method [37] from dried blood spots present on Guthrie Cards collected for neonatal screenings. In order to confine the genetic analyses to individuals of Sardinian origin, we took advantage of the patrilinear transmission of both chromosome Y markers and surnames and performed a survey of the surname distribution of each individual to be analysed using the website GENS [38]. More specifically, samples of individuals with surnames that were more common in continental Italy were not considered in this study. This selection was simplified by the fact that most Sardinians exhibit peculiar surnames, clearly distinct from those encountered in the Italian mainland. This peculiarity is due to the fact that the Sardinian language is differentiated within the romance language family, and hence the derived surnames are unequivocally distinguishable from the non-Sardinian ones. Using these surname based criteria, 0.9% of the chromosome Y samples were eliminated. Because of the sample selection strategy, this study is not addressed to evaluate the effect of very recent genetic flow from outside populations - essentially from the Italian mainland - that, according to demographic data, occurred mainly during the last sixty years. In addition, to ensure independence of the subjects analysed, only individuals born during an eight month sample selection period were included and, in cases of male twins, only one was considered. Using this approach we collected 187, 103 and 86 samples from newborns in delivery units located in Cagliari, Sorgono and Tempio respectively (Figure 1). The sample set from Cagliari includes newborns from the southern and most densely-populated area of Sardinia, the Campidano region. The sample set from Sorgono comprises newborns from the Barbagia area, representing the most internal and isolated area of the island. Until a few decades ago, the population of Barbagia lived in rather strict geographical and cultural isolation, distributed amongst small and dispersed villages. Finally, the sample set from Tempio refers to the Gallura area that is linguistically differentiated from the rest of the island, showing similarities with the dialect spoken on the nearby island of Corsica. Thus, these three regions were selected to maximise the possibility of finding population heterogeneity and to compare regions with different demographic history.

We also considered an additional Sardinian sample set, for the most part from a case-control study, consisting of 155 healthy adult male blood donors from the transfusion centre of the Brotzu Hospital in Cagliari and 399 type 1 diabetes patient samples from a previous study [18]. The present study was approved by the institutional review board of the University of Cagliari. Each participant signed an informed consent form for the samples used. In the case of newborns, consent was obtained from the child's parents.

### Genotyping

We assessed the samples for the SNPs M9, M13, M17, M18, M26, M35, M67, M68, M78, M89, M92, M102, M123, M130, M170, M172, M173, M201 M267 and M269. An Alu insertion (YAP) was used for M1, for a total of 21 Y-chromosome biallelic polymorphisms, all located in the male-specific portion of the Y-chromosome [14], [15], [18], [39]. Lineages defined by the detected SNPs are indicated according to the cladistic name by mutation, following the conventional nomenclature of the Y-Chromosome Consortium (2002) [40]. It should be noted that in the present study, nucleotide deletion and insertion characterising the M17 and M18 haplogroup lineages respectively were genotyped by direct capillary sequencing of all M173 positive samples, since conventional genotyping strategies do not appear to be appropriate in distinguishing between these variants (unpublished data).

The 9 STR loci considered in this study (*DYS19, DYS385a, DYS385b, DYS389-I, DYS389-II, DYS390, DYS391, DYS392, DYS393*) are all tetra-nucleotide repeats, except for the tri-nucleotide repeat *DYS392*, and were part of the Men-type Argus Y-HM PCR amplification kit, developed according to recommendations of the International Forensic Y-User Group [41]. Y-STR loci were genotyped by separating the fluorescent-tagged PCR products on a capillary-sequencer (ABI PRISM 3100 Genetic Analyzer) following the manufacturer's instructions. Note that 3 of the 9 STRs originally typed were excluded from the analyses since two of them (*DYS385a, DYS385b*) were duplicated and phylogenetically indistinguishable (and thus not valid for the tests performed in this study), and another (*DYS392*) provided genotypes of marginal quality below the threshold of acceptance (90%) for inclusion in this study.

### Statistical analysis of the data

We used conventional pairwise F_ST_ (Fixation Index-Statistics), applied on the data from biallelic markers, as a short-term genetic distance measure between different Sardinian and European populations [42]–[44], testing distribution under the null hypothesis of “no genetic differentiation between the populations”, by permuting haplotypes between populations. We also applied pairwise R_ST_, an *F*-statistic derived under a stepwise mutation model to the microsatellite data [45]. Gene diversity and its sampling variance were estimated according to Nei [46], as the probability that two randomly-chosen haplotypes are different in the sample; an estimate equivalent to the expected heterozygosity for diploid data. We computed pairwise conventional F_ST_, R_ST_ and gene diversity using the Arlequin 3.11 software [47], [48]. Pre-processing of datasets for bootstrapping and format conversions was performed using ad hoc Python scripts on a Linux cluster machine. A thousand FST and RST distances were then clustered using PHYLIP (PHYlogeny Inference Package) v3.66 using the UPGMA tree drawing method and unrooted consensus trees were built with the PHYLIP executable “Consense”. Trees were then drawn with the PHYLIP executable “Drawtree” [49], [50]. We carried out full likelihood Bayesian inference of genetic and demographic parameters under population growth using the BATWING programme (Bayesian analysis of trees with internal node generation) [51], [52]. BATWING uses a Markov chain Monte Carlo procedure to generate a sequence of genealogic and population trees, with associated model parameter values, consistent with the genetic data observed in a sample of individuals. At equilibrium, the sequence of trees samples from the posterior probability distribution of trees, given the observed data and the assumed underlying genetic and demographic model. The extended BATWING version used here assumes an unbounded single stepwise mutation model for the microsatellite loci and a coalescent process under an exponential model of population growth from an initially constant-size population. A unique prior for the microsatellite mutation rate was based on the Zhivotovsky et al. [53] estimate as 6.9×10^−4^ and applied to *DYS19, DYS389I, DYS389II, DYS390*, *DYS391*, and *DYS393*, setting gamma as (1.47, 2130) (mean = 0.00069, SD = 0.00057) [54]. In this analysis we avoided using samples containing the duplicated *DYS19* microsatellite. Weakly informative priors were also given other parameters to aid convergence of the MCMC process. The initial effective population size was given a gamma (1, 0.0001) (mean = 10,000, SD = 10,000), which covers the values commonly assumed for the global Y-chromosome effective population size as well as lower values to compensate for this being a regional sample and for representing the effective size before growth [55]. Population growth rate alpha per generation was given a gamma (2, 400) (mean = 0.005, SD = 0.0035). This very flat prior gives support to much lower alpha values and covers estimates of real (census) population growth in various parts of the world over the past few thousand years [56] as well as supporting lower values to allow for growth in effective population size being plausibly lower than real growth. The time in coalescent units when exponential growth began, or Beta, was given a gamma (2, 1) (mean = 2, SD = 1.41) [51]. All other parameters were given flat, uninformative priors [55] Generation time was set at 25 years as used elsewhere in Y-chromosome studies [57]–[59], adapting the estimated generation times for present day males to the presumably shorter life span in the past [60]. Although a natural measure of the central tendency of a sample of continuous data is its mode (the most probable value), the mean and median are the most popular measures of location due to their simplicity and ease of estimation. The median is often used instead of the mean for asymmetric data because it is closer to the mode and is insensitive to extreme values in the sample.

However, the distribution of our simulated data appears definitely skewed, non-normal and exposed to unpredictable contamination depending on the Markov Chain Monte Carlo sampling of the state space. In this condition, mean and median exhibit remarkable bias whereas the mode can be reliably estimated by several robust estimators [61]. In this manuscript, after verifying that none of the markers examined showed bi-modal distribution, we employed the Half-Range Mode (HRM). This is based on subsequent subdivision of the data set in an iterative fashion and appears to be one of the best compromises between reliability, ease of implementation and computing time. Indeed HRM is robust for a wide variety of distribution and contamination levels [62]. This mode estimate also seems less affected by the sample size effect observed with mean-based estimates since, in our data, good convergence for the mode could be obtained even with relatively small sampling (∼10^7^), resulting in a good compromise between accuracy of calculation and computation time.

## Supporting Information

Table S1 STRs typed in the three Sardinian subpopulations
(0.58 MB DOC)

Table S2 Conventional Population Pairwise FST values between different European populations
(0.06 MB DOC)

Table S3 FST P values. Number of permutations: 10100
(0.06 MB DOC)

Table S4 Pairwise squared size difference RST values between different European populations
(0.06 MB DOC)

Table S5 RST P values. Number of permutations: 10100
(0.06 MB DOC)

Table S6 AMOVA analysis of 12 pre-molecular markers on 21 linguistic domains of Sardinia
(0.05 MB DOC)

## References

[1] Siniscalco M, Bernini L, Latte B, Motulsky AG (1961). Favism and Thalassæmia in Sardinia and their Relationship to Malaria.. Nature.

[2] Rosatelli MC, Dozy A, Faa V, Meloni A, Sardu R (1992). Molecular characterization of beta-thalassemia in the Sardinian population.. Am J Hum Genet.

[3] Loudianos G, Dessi V, Lovicu M, Angius A, Figus A (1999). Molecular characterization of wilson disease in the Sardinian population–evidence of a founder effect.. Hum Mutat.

[4] Rosatelli MC, Meloni A, Devoto M, Cao A, Scott HS (1998). A common mutation in Sardinian autoimmune polyendocrinopathy- candidiasis-ectodermal dystrophy patients.. Hum Genet.

[5] Karvonen M, Tuomilehto J, Libman I, La Porte R (1993). A review of the recent epidemiological data on the worldwide incidence of type 1 (insulin-dependent) diabetes mellitus. World Health Organization DIAMOND Project Group.. Diabetologia.

[6] Pugliatti M, Rosati G, Carton H, Riise T, Drulovic J (2006). The epidemiology of multiple sclerosis in Europe.. Eur J Neurol.

[7] Lilliu G (1982). La civiltà nuragica..

[8] Lampis R, Morelli L, Congia M, Macis MD, Mulargia A (2000). The inter-regional distribution of HLA class II haplotypes indicates the suitability of the Sardinian population for case-control association studies in complex diseases.. Hum Mol Genet.

[9] Contu L, Arras M, Carcassi C, La Nasa G, Mulargia M (1992). HLA structure of the Sardinian population: a haplotype study of 551 families.. Tissue Antigens.

[10] Zei G, Lisa A, Fiorani O, Magri C, Quintana-Murci L (2003). From surnames to the history of Y chromosomes: the Sardinian population as a paradigm.. Eur J Hum Genet.

[11] Scozzari R, Cruciani F, Pangrazio A, Santolamazza P, Vona G (2001). Human Y-chromosome variation in the western Mediterranean area: implications for the peopling of the region.. Hum Immunol.

[12] Cappello N, Rendine S, Griffo R, Mameli GE, Succa V (1996). Genetic analysis of Sardinia: I. data on 12 polymorphisms in 21 linguistic domains.. Ann Hum Genet.

[13] Fraumene C, Petretto E, Angius A, Pirastu M (2003). Striking differentiation of sub-populations within a genetically homogeneous isolate (Ogliastra) in Sardinia as revealed by mtDNA analysis.. Hum Genet.

[14] Semino O, Passarino G, Oefner PJ, Lin AA, Arbuzova S (2000). The genetic legacy of Paleolithic Homo sapiens sapiens in extant Europeans: a Y chromosome perspective.. Science.

[15] Underhill PA, Shen P, Lin AA, Jin L, Passarino G (2000). Y chromosome sequence variation and the history of human populations.. Nat Genet.

[16] Bosch E, Calafell F, Comas D, Oefner PJ, Underhill PA (2001). High-resolution analysis of human Y-chromosome variation shows a sharp discontinuity and limited gene flow between northwestern Africa and the Iberian Peninsula.. Am J Hum Genet.

[17] Wells RS, Yuldasheva N, Ruzibakiev R, Underhill PA, Evseeva I (2001). The Eurasian heartland: a continental perspective on Y-chromosome diversity.. Proc Natl Acad Sci U S A.

[18] Contu D, Morelli L, Zavattari P, Lampis R, Angius E (2002). Sex-related bias and exclusion mapping of the nonrecombinant portion of chromosome Y in human type 1 diabetes in the isolated founder population of Sardinia.. Diabetes.

[19] Francalacci P, Morelli L, Underhill PA, Lillie AS, Passarino G (2003). Peopling of three Mediterranean islands (Corsica, Sardinia, and Sicily) inferred by Y-chromosome biallelic variability.. Am J Phys Anthropol.

[20] Cinnioglu C, King R, Kivisild T, Kalfoglu E, Atasoy S (2004). Excavating Y-chromosome haplotype strata in Anatolia.. Hum Genet.

[21] Capelli C, Redhead N, Abernethy JK, Gratrix F, Wilson JF (2003). A Y chromosome census of the British Isles.. Curr Biol.

[22] Bosch E, Calafell F, Rosser ZH, Norby S, Lynnerup N (2003). High level of male-biased Scandinavian admixture in Greenlandic Inuit shown by Y-chromosomal analysis.. Hum Genet.

[23] Rootsi S, Magri C, Kivisild T, Benuzzi G, Help H (2004). Phylogeography of Y-chromosome haplogroup I reveals distinct domains of prehistoric gene flow in europe.. Am J Hum Genet.

[24] Bosch E, Calafell F, Gonzalez-Neira A, Flaiz C, Mateu E (2006). Paternal and maternal lineages in the Balkans show a homogeneous landscape over linguistic barriers, except for the isolated Aromuns.. Ann Hum Genet.

[25] Sondaar PY, Elburg R, Klein-Hofmeijer G, Martini F, Sanges M (1995). The human colonization of Sardinia: a Late-Pleistocene human fossil from Corbeddu cave.. Comptes Rendus de l'Académie des Sciences Paris.

[26] Sondaar PY, Balmuth MS, Tykot RH (1998). Palaeolithic Sardinians: paleontological evidence and methods.. Sardinian and Aegean chronology.

[27] Torroni A, Bandelt HJ, Macaulay V, Richards M, Cruciani F (2001). A signal, from human mtDNA, of postglacial recolonization in Europe.. Am J Hum Genet.

[28] Achilli A, Rengo C, Magri C, Battaglia V, Olivieri A (2004). The molecular dissection of mtDNA haplogroup H confirms that the Franco-Cantabrian glacial refuge was a major source for the European gene pool.. Am J Hum Genet.

[29] Lappalainen T, Koivumaki S, Salmela E, Huoponen K, Sistonen P (2006). Regional differences among the Finns: a Y-chromosomal perspective.. Gene.

[30] Helgason A, Yngvadottir B, Hrafnkelsson B, Gulcher J, Stefansson K (2005). An Icelandic example of the impact of population structure on association studies.. Nat Genet.

[31] Angius A, Bebbere D, Petretto E, Falchi M, Forabosco P (2002). Not all isolates are equal: linkage disequilibrium analysis on Xq13.3 reveals different patterns in Sardinian sub-populations.. Hum Genet.

[32] Zavattari P, Deidda E, Whalen M, Lampis R, Mulargia A (2000). Major factors influencing linkage disequilibrium by analysis of different chromosome regions in distinct populations: demography, chromosome recombination frequency and selection.. Hum Mol Genet.

[33] Flores C, Maca-Meyer N, Gonzalez AM, Oefner PJ, Shen P (2004). Reduced genetic structure of the Iberian peninsula revealed by Y-chromosome analysis: implications for population demography.. Eur J Hum Genet.

[34] Cucca F, Lampis R, Frau F, Macis D, Angius E (1995). The distribution of DR4 haplotypes in Sardinia suggests a primary association of insulin dependent diabetes mellitus with DRB1 and DQB1 loci.. Hum Immunol.

[35] Marrosu MG, Murru R, Costa G, Melis MC, Rolesu M (2007). Variation of the myelin oligodendrocyte glycoprotein gene is not primarily associated with multiple sclerosis in the Sardinian population.. BMC Genet.

[36] Zoledziewska M, Perra C, Orru V, Moi L, Frongia P (2007). Further evidence of a primary, causal association of the PTPN22 620W variant with type 1 diabetes.. Diabetes..

[37] Walsh PS, Metzger DA, Higuchi R (1991). Chelex 100 as a medium for simple extraction of DNA for PCR-based typing from forensic material.. Biotechniques.

[38] http://gens.labo.net

[39] Underhill PA, Jin L, Lin AA, Mehdi SQ, Jenkins T (1997). Detection of numerous Y chromosome biallelic polymorphisms by denaturing high-performance liquid chromatography.. Genome Res.

[40] Y-Chromosome-Consortium (2002). A nomenclature system for the tree of human Y-chromosomal binary haplogroups.. Genome Res.

[41] http://www.ystr.org/index.html

[42] Dempster A, Laird N, Rubin D (1977). Maximum likelihood estimation from incomplete data via the EM algorithm.. Journal of the Royal Statistical Society.

[43] Excoffier L, Slatkin M (1995). Maximum-likelihood estimation of molecular haplotype frequencies in a diploid population.. Mol Biol Evol.

[44] Lange K (1997). Mathematical and statistical methods for genetic analysis..

[45] Slatkin M (1995). A measure of population subdivision based on microsatellite allele frequencies.. Genetics.

[46] Nei M (1987). Molecular evolutionary genetics..

[47] http://cmpg.unibe.ch/software/arlequin3/

[48] Excoffier L, Laval G, Schneider S (2005). An integrated software package for population genetics data analysis.. Evolutionary Bioinformatics Online.

[49] http://evolution.genetics.washington.edu/phylip.html.

[50] Felsestein J (1989). PHYLIP-Phylogeny Inference Package (Version 3.2).. Cladistics.

[51] Wilson IJW, M.E.;, Balding DJ (2003). Inferences from DNA data: population histories, evolutionary processes and forensic match probabilities.. J R Statist Soc A.

[52] http://www.mas.ncl.ac.uk/nijw/

[53] Zhivotovsky LA, Underhill PA, Cinnioglu C, Kayser M, Morar B (2004). The effective mutation rate at Y chromosome short tandem repeats, with application to human population-divergence time.. Am J Hum Genet.

[54] Xue Y, Zerjal T, Bao W, Zhu S, Shu Q (2006). Male demography in East Asia: a north-south contrast in human population expansion times.. Genetics.

[55] Weale ME, Weiss DA, Jager RF, Bradman N, Thomas MG (2002). Y chromosome evidence for Anglo-Saxon mass migration.. Mol Biol Evol.

[56] Cavalli-Sforza LLM, P.;, Piazza A (1994). The History and Geography of Human Genes..

[57] Thomson R, Pritchard JK, Shen P, Oefner PJ, Feldman MW (2000). Recent common ancestry of human Y chromosomes: evidence from DNA sequence data.. Proc Natl Acad Sci U S A.

[58] Rootsi S, Zhivotovsky LA, Baldovic M, Kayser M, Kutuev IA (2007). A counter-clockwise northern route of the Y-chromosome haplogroup N from Southeast Asia towards Europe.. Eur J Hum Genet.

[59] Martinez L, Underhill PA, Zhivotovsky LA, Gayden T, Moschonas NK (2007). Paleolithic Y-haplogroup heritage predominates in a Cretan highland plateau.. Eur J Hum Genet.

[60] Jobling MA, Tyler-Smith C (2003). The human Y chromosome: an evolutionary marker comes of age.. Nat Rev Genet.

[61] Hedges SB, Shah P (2003). Comparison of mode estimation methods and application in molecular clock analysis.. BMC Bioinformatics.

[62] Bickel DR (2002). Robust estimators of the mode and skewness of continuous data.. Comp Stat Data Anal.

