# Supplementary material for: Y-Chromosome Based Evidence for Pre-Neolithic Origin of the Genetically Homogeneous but Diverse Sardinian Population: Inference for Association Scans
Source: PLoS One. 2008 Jan 9;3(1):e1430. doi: 10.1371/journal.pone.0001430 (PMC2174525; doi:10.1371/journal.pone.0001430)
Supplement: Table S1 — STRs typed in the three Sardinian subpopulations (0.58 MB DOC) [file pone.0001430.s001.doc]

Table S1. STRs typed in the three Sardinian subpopulations

| **Population** | **Haplotype (DYS)** | **19** | **385I** | **385II** | **389I** | **389II** | **390** | **391** | **393** |
| --- | --- | --- | --- | --- | --- | --- | --- | --- | --- |
| Cagliari | M1 | 16 | 16 | 16 | 11 | 29 | 22 | 9 | 13 |
| Cagliari | M1, M35 | 13 | 13 | 14 | 14 | 31 | 24 | 9 | 13 |
| Cagliari | M1, M35, M123 | 13 | 15 | 16 | 13 | 31 | 24 | 10 | 12 |
| Cagliari | M1, M35, M123 | 13 | 15 | 16 | 13 | 30 | 24 | 10 | 13 |
| Cagliari | M1, M35, M123 | 12 | 16 | 16 | 13 | 32 | 26 | 10 | 13 |
| Cagliari | M1, M35, M78 | 13 | 16 | 17 | 14 | 30 | 24 | 10 | 13 |
| Cagliari | M1, M35, M78 | 14 | 16 | 18 | 13 | 30 | 25 | 10 | 13 |
| Cagliari | M1, M35, M78 | 13 | 16 | 19 | 13 | 30 | 24 | 10 | 13 |
| Cagliari | M1, M35, M78 | 13 | 16 | 18 | 13 | 30 | 24 | 10 | 13 |
| Cagliari | M1, M35, M78 | 14 | 18 | 19 | 13 | 30 | 24 | 10 | 13 |
| Cagliari | M1, M35, M78 | 13 | 16 | 19 | 13 | 30 | 24 | 10 | 13 |
| Cagliari | M1, M35, M78 | 15 | 18 | 19 | 13 | 30 | 24 | 10 | 13 |
| Cagliari | M1, M35, M78 | 13 | 15 | 18 | 13 | 30 | 24 | 10 | 13 |
| Cagliari | M1, M35, M78 | 13 | 16 | 18 | 13 | 30 | 24 | 10 | 14 |
| Cagliari | M1, M35, M78 | 13 | 16 | 18 | 13 | 30 | 24 | 10 | 13 |
| Cagliari | M13 | 15 | 11 | 12 | 12 | 28 | 22 | 10 | 13 |
| Cagliari | M89, M170 | 15 | 12 | 13 | 13 | 30 | 24 | 10 | 14 |
| Cagliari | M89, M170 | 15 | 12 | 13 | 13 | 30 | 24 | 10 | 14 |
| Cagliari | M89, M170 | 14 | 13 | 19 | 13 | 30 | 23 | 10 | 12 |
| Cagliari | M89, M170 | 15 | 12 | 13 | 13 | 30 | 24 | 10 | 14 |
| Cagliari | M89, M170 | 15 | 15 | 15 | 13 | 32 | 23 | 10 | 15 |
| Cagliari | M89, M170 | 15 | 13 | 16 | 13 | 31 | 23 | 10 | 14 |
| Cagliari | M89, M170, M26 | 17 | 12 | 12 | 13 | 28 | 23 | 10 | 14 |
| Cagliari | M89, M170, M26 | 17 | 12 | 13 | 13 | 28 | 23 | 10 | 13 |
| Cagliari | M89, M170, M26 | 17 | 12 | 12 | 14 | 29 | 24 | 10 | 13 |
| Cagliari | M89, M170, M26 | 17 | 13 | 13 | 13 | 28 | 24 | 10 | 13 |
| Cagliari | M89, M170, M26 | 15 | 12 | 12 | 13 | 28 | 23 | 10 | 13 |
| Cagliari | M89, M170, M26 | 17 | 12 | 13 | 13 | 28 | 23 | 10 | 13 |
| Cagliari | M89, M170, M26 | 16 | 12 | 12 | 13 | 28 | 22 | 10 | 13 |
| Cagliari | M89, M170, M26 | 16 | 12 | 13 | 13 | 28 | 23 | 10 | 14 |
| Cagliari | M89, M170, M26 | 17 | 12 | 12 | 12 | 28 | 23 | 10 | 13 |
| Cagliari | M89, M170, M26 | 17 | 12 | 13 | 13 | 28 | 23 | 10 | 13 |
| Cagliari | M89, M170, M26 | 17 | 12 | 13 | 13 | 28 | 23 | 10 | 13 |
| Cagliari | M89, M170, M26 | 14 | 11 | 14 | 14 | 30 | 23 | 11 | 13 |
| Cagliari | M89, M170, M26 | 15 | 12 | 12 | 13 | 27 | 23 | 10 | 13 |
| Cagliari | M89, M170, M26 | 17 | 12 | 15 | 14 | 29 | 23 | 10 | 13 |
| Cagliari | M89, M170, M26 | 17 | 12 | 12 | 13 | 28 | 23 | 10 | 12 |
| Cagliari | M89, M170, M26 | 17 | 13 | 13 | 12 | 27 | 23 | 11 | 13 |
| Cagliari | M89, M170, M26 | 16 | 11 | 12 | 13 | 28 | 23 | 10 | 13 |
| Cagliari | M89, M170, M26 | 16 | 12 | 12 | 13 | 30 | 23 | 10 | 14 |
| Cagliari | M89, M170, M26 | 17 | 11 | 13 | 13 | 28 | 24 | 10 | 13 |
| Cagliari | M89, M170, M26 | 17 | 11 | 12 | 13 | 28 | 23 | 10 | 13 |
| Cagliari | M89, M170, M26 | 16 | 12 | 12 | 13 | 28 | 22 | 10 | 13 |
| Cagliari | M89, M170, M26 | 16 | 11 | 14 | 13 | 28 | 24 | 10 | 13 |
| Cagliari | M89, M170, M26 | 17 | 11 | 13 | 13 | 28 | 24 | 10 | 12 |
| Cagliari | M89, M170, M26 | 16 | 12 | 14 | 13 | 29 | 23 | 10 | 13 |
| Cagliari | M89, M170, M26 | 16 | 13 | 13 | 13 | 28 | 23 | 10 | 9 |
| Cagliari | M89, M170, M26 | 16 | 11 | 11 | 14 | 29 | 24 | 10 | 13 |
| Cagliari | M89, M170, M26 | 17 | 12 | 12 | 13 | 28 | 23 | 10 | 13 |
| Cagliari | M89, M170, M26 | 16 | 12 | 13 | 14 | 29 | 23 | 10 | 13 |
| Cagliari | M89, M170, M26 | 17 | 12 | 13 | 13 | 28 | 24 | 10 | 13 |
| Cagliari | M89, M170, M26 | 16 | 12 | 12 | 13 | 28 | 21 | 10 | 13 |
| Cagliari | M89, M170, M26 | 15 | 11 | 13 | 13 | 28 | 24 | 10 | 13 |
| Cagliari | M89, M170, M26 | 17 | 12 | 12 | 13 | 28 | 23 | 10 | 13 |
| Cagliari | M89, M170, M26 | 15 | 12 | 12 | 13 | 28 | 23 | 10 | 13 |
| Cagliari | M89, M170, M26 | 17 | 12 | 12 | 13 | 28 | 23 | 10 | 13 |
| Cagliari | M89, M170, M26 | 14 | 11 | 14 | 14 | 30 | 23 | 11 | 13 |
| Cagliari | M89, M170, M26 | 16 | 12 | 12 | 14 | 31 | 23 | 10 | 14 |
| Cagliari | M89, M170, M26 | 17 | 12 | 13 | 13 | 28 | 23 | 10 | 13 |
| Cagliari | M89, M170, M26 | 16 | 12 | 13 | 14 | 29 | 23 | 10 | 13 |
| Cagliari | M89, M170, M26 | 15 | 11 | 12 | 13 | 29 | 23 | 10 | 13 |
| Cagliari | M89, M170, M26 | 15 | 12 | 12 | 13 | 28 | 23 | 10 | 13 |
| Cagliari | M89, M170, M26 | 17 | 12 | 12 | 13 | 29 | 23 | 10 | 13 |
| Cagliari | M89, M170, M26 | 16 | 12 | 13 | 14 | 29 | 23 | 10 | 13 |
| Cagliari | M89, M170, M26 | 15 | 12 | 12 | 13 | 28 | 23 | 10 | 13 |
| Cagliari | M89, M170, M26 | 17 | 11 | 12 | 14 | 29 | 23 | 10 | 13 |
| Cagliari | M89, M170, M26 | 17 | 12 | 13 | 13 | 28 | 23 | 10 | 13 |
| Cagliari | M89, M170, M26 | 17 | 12 | 12 | 13 | 28 | 23 | 10 | 13 |
| Cagliari | M89, M170, M26 | 15 | 12 | 13 | 13 | 29 | 24 | 10 | 13 |
| Cagliari | M89, M170, M26 | 16 | 12 | 12 | 14 | 29 | 23 | 10 | 13 |
| Cagliari | M89, M170, M26 | 16 | 12 | 12 | 13 | 30 | 23 | 10 | 13 |
| Cagliari | M89, M170, M26 | 17 | 12 | 12 | 13 | 28 | 23 | 10 | 13 |
| Cagliari | M89, M170, M26 | 15 | 12 | 12 | 14 | 29 | 23 | 10 | 13 |
| Cagliari | M89, M170, M26 | 16 | 12 | 12 | 13 | 30 | 24 | 10 | 13 |
| Cagliari | M89, M170, M26 | 16 | 12 | 12 | 13 | 28 | 23 | 10 | 13 |
| Cagliari | M89, M170, M26 | 17 | 12 | 12 | 13 | 28 | 23 | 10 | 13 |
| Cagliari | M89, M170, M26 | 16 | 12 | 13 | 13 | 28 | 23 | 10 | 13 |
| Cagliari | M89, M172 | 15 | 16 | 16 | 13 | 29 | 23 | 10 | 14 |
| Cagliari | M89, M172 | 14 | 14 | 17 | 13 | 29 | 24 | 10 | 13 |
| Cagliari | M89, M172 | 14 | 13 | 16 | 13 | 29 | 24 | 11 | 12 |
| Cagliari | M89, M172 | 14 16 | 13 | 16 | 14 | 29 | 23 | 10 | 12 |
| Cagliari | M89, M172, M102 | 15 | 14 | 17 | 12 | 27 | 24 | 10 | 12 |
| Cagliari | M89, M172, M102 | 15 | 14 | 17 | 12 | 28 | 23 | 10 | 12 |
| Cagliari | M89, M172, M102 | 15 | 15 | 17 | 13 | 29 | 24 | 10 | 12 |
| Cagliari | M89, M172, M102 | 15 | 15 | 15 | 12 | 28 | 24 | 10 | 12 |
| Cagliari | M89, M172, M102 | 15 | 13 | 16 | 12 | 28 | 23 | 10 | 12 |
| Cagliari | M89, M172, M67 | 14 | 13 | 13 | 13 | 29 | 23 | 10 | 12 |
| Cagliari | M89, M172, M67 | 15 | 12 | 15 | 13 | 29 | 23 | 10 | 12 |
| Cagliari | M89, M172, M67 | 14 | 12 | 12 | 14 | 31 | 23 | 10 | 12 |
| Cagliari | M89, M172, M67 | 14 | 13 | 16 | 13 | 30 | 23 | 10 | 12 |
| Cagliari | M89, M172, M67 | 15 | 13 | 16 | 13 | 29 | 23 | 10 | 12 |
| Cagliari | M89, M172, M67 | 14 | 13 | 19 | 13 | 30 | 24 | 10 | 12 |
| Cagliari | M89, M172, M67 | 14 | 14 | 17 | 13 | 31 | 23 | 10 | 12 |
| Cagliari | M89, M172, M67, M92 | 15 | 13 | 14 | 13 | 29 | 22 | 10 | 13 |
| Cagliari | M89, M172, M67, M92 | 15 | 13 | 14 | 13 | 27 | 23 | 10 | 13 |
| Cagliari | M89, M172, M67, M92 | 15 | 13 | 15 | 13 | 29 | 22 | 10 | 13 |
| Cagliari | M89, M201 | 15 | 14 | 15 | 12 | 29 | 22 | 10 | 14 |
| Cagliari | M89, M201 | 15 | 14 | 15 | 14 | 31 | 23 | 11 | 14 |
| Cagliari | M89, M201 | 15 | 15 | 15 | 13 | 31 | 23 | 10 | 14 |
| Cagliari | M89, M201 | 15 | 11 | 14 | 14 | 30 | 22 | 10 | 13 |
| Cagliari | M89, M201 | 15 | 13 | 17 | 12 | 29 | 24 | 10 | 14 |
| Cagliari | M89, M201 | 15 | 13 | 14 | 13 | 30 | 22 | 10 | 14 |
| Cagliari | M89, M201 | 15 | 13 | 15 | 12 | 29 | 21 | 10 | 14 |
| Cagliari | M89, M201 | 15 | 15 | 16 | 12 | 29 | 23 | 10 | 14 |
| Cagliari | M89, M201 | 16 | 14 | 15 | 12 | 29 | 23 | 10 | 14 |
| Cagliari | M89, M201 | 15 | 13 | 14 | 12 | 29 | 23 | 10 | 13 |
| Cagliari | M89, M201 | 15 | 15 | 16 | 12 | 29 | 23 | 10 | 14 |
| Cagliari | M89, M201 | 15 | 13 | 15 | 11 | 28 | 23 | 10 | 14 |
| Cagliari | M89, M201 | 15 | 11 | 14 | 13 | 30 | 22 | 11 | 14 |
| Cagliari | M89, M201 | 15 | 15 | 16 | 12 | 28 | 22 | 9 | 14 |
| Cagliari | M89, M201 | 15 | 11 | 14 | 14 | 30 | 22 | 10 | 13 |
| Cagliari | M89, M201 | 15 | 11 | 14 | 14 | 30 | 22 | 10 | 13 |
| Cagliari | M89, M201 | 15 | 14 | 15 | 12 | 29 | 22 | 10 | 14 |
| Cagliari | M89, M201 | 15 16 | 13 | 14 | 13 | 31 | 22 | 11 | 14 |
| Cagliari | M89, M201 | 15 | 11 | 14 | 13 | 29 | 22 | 11 | 13 |
| Cagliari | M89, M201 | 15 | 13 | 15 | 12 | 29 | 21 | 10 | 14 |
| Cagliari | M89, M201 | 15 | 13 | 15 | 11 | 28 | 23 | 10 | 14 |
| Cagliari | M89, M201 | 15 | 12 | 14 | 13 | 31 | 22 | 10 | 13 |
| Cagliari | M89, M201 | 15 | 15 | 15 | 12 | 29 | 23 | 10 | 14 |
| Cagliari | M89, M201 | 15 | 11 | 14 | 13 | 30 | 22 | 11 | 14 |
| Cagliari | M89, M201 | 15 | 14 | 14 | 12 | 27 | 23 | 10 | 13 |
| Cagliari | M89, M201 | 15 | 12 | 15 | 12 | 29 | 22 | 10 | 14 |
| Cagliari | M89, M267 | 14 | 13 | 19 | 13 | 30 | 23 | 10 | 12 |
| Cagliari | M89, M267 | 14 | 13 | 19 | 13 | 30 | 23 | 11 | 12 |
| Cagliari | M89, M267 | 14 | 13 | 19 | 14 | 31 | 23 | 10 | 12 |
| Cagliari | M89, M267 | 14 | 13 | 21 | 14 | 31 | 23 | 10 | 12 |
| Cagliari | M89, M267 | 14 | 11 | 19 | 13 | 29 | 22 | 11 | 13 |
| Cagliari | M89, M9 | 15 | 15 | 16 | 13 | 29 | 23 | 11 | 13 |
| Cagliari | M89, M9 | 15 | 13 | 15 | 12 | 29 | 21 | 10 | 14 |
| Cagliari | M89, M9, M173 | 15 | 14 | 14 | 14 | 31 | 23 | 11 | 13 |
| Cagliari | M89, M9, M173 | 15 | 14 | 14 | 14 | 31 | 23 | 11 | 13 |
| Cagliari | M89, M9, M173 | 15 | 14 | 15 | 14 | 31 | 23 | 11 | 13 |
| Cagliari | M89, M9, M173 | 15 | 14 | 14 | 14 | 31 | 23 | 11 | 13 |
| Cagliari | M89, M9, M173, M17 | 14 | 10 | 15 | 13 | 31 | 25 | 10 | 14 |
| Cagliari | M89, M9, M173, M18 | 15 | 12 | 12 | 13 | 28 | 24 | 10 | 13 |
| Cagliari | M89, M9, M173, M18 | 14 | 11 | 14 | 13 | 29 | 23 | 11 | 13 |
| Cagliari | M89, M9, M173, M18 | 15 | 10 | 12 | 13 | 28 | 24 | 10 | 13 |
| Cagliari | M89, M9, M173, M269 | 14 | 11 | 15 | 12 | 28 | 24 | 11 | 13 |
| Cagliari | M89, M9, M173, M269 | 14 | 11 | 14 | 13 | 29 | 24 | 11 | 13 |
| Cagliari | M89, M9, M173, M269 | 14 | 11 | 15 | 13 | 29 | 24 | 10 | 14 |
| Cagliari | M89, M9, M173, M269 | 14 | 11 | 14 | 13 | 29 | 24 | 11 | 13 |
| Cagliari | M89, M9, M173, M269 | 14 | 11 | 14 | 13 | 29 | 23 | 11 | 13 |
| Cagliari | M89, M9, M173, M269 | 14 | 11 | 14 | 14 | 30 | 24 | 11 | 13 |
| Cagliari | M89, M9, M173, M269 | 14 | 11 | 14 | 13 | 29 | 25 | 11 | 14 |
| Cagliari | M89, M9, M173, M269 | 14 | 11 | 13,2 | 14 | 31 | 24 | 11 | 13 |
| Cagliari | M89, M9, M173, M269 | 14 | 11 | 15 | 13 | 29 | 24 | 11 | 13 |
| Cagliari | M89, M9, M173, M269 | 14 | 11 | 14 | 14 | 30 | 23 | 11 | 14 |
| Cagliari | M89, M9, M173, M269 | 14 | 10 | 15 | 13 | 29 | 23 | 10 | 13 |
| Cagliari | M89, M9, M173, M269 | 14 | 11 | 14 | 14 | 30 | 23 | 11 | 13 |
| Cagliari | M89, M9, M173, M269 | 15 | 11 | 14 | 13 | 29 | 23 | 10 | 13 |
| Cagliari | M89, M9, M173, M269 | 14 | 11 | 14 | 13 | 30 | 24 | 10 | 13 |
| Cagliari | M89, M9, M173, M269 | 14 | 10 | 14 | 14 | 30 | 24 | 10 | 12 |
| Cagliari | M89, M9, M173, M269 | 14 | 11 | 14 | 13 | 29 | 24 | 11 | 13 |
| Cagliari | M89, M9, M173, M269 | 14 | 12 | 14 | 13 | 29 | 25 | 10 | 12 |
| Cagliari | M89, M9, M173, M269 | 14 | 10 | 15 | 13 | 29 | 24 | 10 | 13 |
| Cagliari | M89, M9, M173, M269 | 14 | 11 | 14 | 14 | 30 | 23 | 11 | 13 |
| Cagliari | M89, M9, M173, M269 | 14 | 11 | 14 | 15 | 31 | 23 | 11 | 13 |
| Cagliari | M89, M9, M173, M269 | 14 | 11 | 11 | 13 | 29 | 21 | 11 | 13 |
| Cagliari | M89, M9, M173, M269 | 14 | 11 | 14 | 14 | 30 | 23 | 11 | 13 |
| Cagliari | M89, M9, M173, M269 | 14 | 11 | 14 | 13 | 29 | 24 | 11 | 13 |
| Cagliari | M89, M9, M173, M269 | 14 | 11 | 15 | 13 | 29 | 21 | 11 | 13 |
| Cagliari | M89, M9, M173, M269 | 14 | 12 | 14 | 13 | 29 | 24 | 10 | 14 |
| Cagliari | M89, M9, M173, M269 | 14 | 11 | 14 | 14 | 30 | 23 | 11 | 13 |
| Cagliari | M89, M9, M173, M269 | 14 | 12 | 14 | 13 | 29 | 25 | 10 | 12 |
| Cagliari | M89, M9, M173, M269 | 14 | 11 | 14 | 13 | 29 | 24 | 10 | 13 |
| Cagliari | M89, M9, M173, M269 | 14 | 11 | 14 | 13 | 29 | 24 | 10 | 12 |
| Sorgono | M1, M35 | 13 | 13 | 14 | 14 | 31 | 24 | 9 | 13 |
| Sorgono | M1, M35, M123 | 13 | 16 | 16 | 13 | 33 | 25 | 11 | 13 |
| Sorgono | M1, M35, M78 | 13 | 16 | 19 | 13 | 30 | 24 | 10 | 13 |
| Sorgono | M1, M35, M78 | 14 | 16 | 19 | 14 | 31 | 23 | 10 | 14 |
| Sorgono | M1, M35, M78 | 13 | 16 | 19 | 13 | 30 | 24 | 10 | 13 |
| Sorgono | M89, M170, M26 | 15 | 12 | 14 | 14 | 29 | 23 | 11 | 14 |
| Sorgono | M89, M170, M26 | 16 | 12 | 13 | 13 | 28 | 23 | 10 | 13 |
| Sorgono | M89, M170, M26 | 16 | 12 | 13 | 13 | 28 | 23 | 10 | 13 |
| Sorgono | M89, M170, M26 | 17 | 12 | 13 | 14 | 30 | 23 | 10 | 13 |
| Sorgono | M89, M170, M26 | 16 | 12 | 12 | 13 | 28 | 23 | 10 | 13 |
| Sorgono | M89, M170, M26 | 16 | 12 | 13 | 13 | 28 | 23 | 10 | 13 |
| Sorgono | M89, M170, M26 | 16 | 12 | 13 | 13 | 28 | 23 | 10 | 13 |
| Sorgono | M89, M170, M26 | 17 | 12 | 12 | 13 | 28 | 21 | 10 | 14 |
| Sorgono | M89, M170, M26 | 16 | 11 | 12 | 12 | 27 | 23 | 10 | 13 |
| Sorgono | M89, M170, M26 | 17 | 11 | 13 | 13 | 28 | 24 | 10 | 13 |
| Sorgono | M89, M170, M26 | 16 | 12 | 13 | 12 | 27 | 23 | 11 | 13 |
| Sorgono | M89, M170, M26 | 17 | 12 | 13 | 14 | 29 | 24 | 10 | 13 |
| Sorgono | M89, M170, M26 | 16 | 12 | 13 | 12 | 27 | 23 | 10 | 13 |
| Sorgono | M89, M170, M26 | 16 | 12 | 12 | 13 | 28 | 23 | 10 | 13 |
| Sorgono | M89, M170, M26 | 17 | 12 | 13 | 13 | 28 | 23 | 10 | 9 |
| Sorgono | M89, M170, M26 | 16 | 12 | 13 | 13 | 28 | 23 | 10 | 13 |
| Sorgono | M89, M170, M26 | 16 | 12 | 13 | 13 | 28 | 23 | 10 | 13 |
| Sorgono | M89, M170, M26 | 17 | 12 | 13 | 12 | 27 | 23 | 10 | 13 |
| Sorgono | M89, M170, M26 | 16 | 11 | 13 | 13 | 28 | 24 | 10 | 13 |
| Sorgono | M89, M170, M26 | 16 | 12 | 12 | 13 | 28 | 23 | 10 | 13 |
| Sorgono | M89, M170, M26 | 15 | 12 | 13 | 13 | 28 | 23 | 10 | 13 |
| Sorgono | M89, M170, M26 | 17 | 11 | 13 | 13 | 28 | 24 | 10 | 13 |
| Sorgono | M89, M170, M26 | 17 | 12 | 13 | 13 | 28 | 23 | 10 | 13 |
| Sorgono | M89, M170, M26 | 16 | 12 | 12 | 13 | 28 | 23 | 10 | 13 |
| Sorgono | M89, M170, M26 | 16 | 12 | 12 | 13 | 28 | 23 | 10 | 13 |
| Sorgono | M89, M170, M26 | 17 | 13 | 13 | 13 | 28 | 23 | 10 | 13 |
| Sorgono | M89, M170, M26 | 16 | 12 | 13 | 13 | 28 | 23 | 10 | 13 |
| Sorgono | M89, M170, M26 | 16 | 12 | 13 | 13 | 28 | 23 | 10 | 13 |
| Sorgono | M89, M170, M26 | 17 | 13 | 13 | 13 | 28 | 23 | 10 | 13 |
| Sorgono | M89, M170, M26 | 16 | 12 | 13 | 13 | 28 | 23 | 10 | 13 |
| Sorgono | M89, M170, M26 | 16 | 12 | 13 | 13 | 28 | 23 | 10 | 13 |
| Sorgono | M89, M170, M26 | 16 | 12 | 12 | 13 | 28 | 23 | 10 | 13 |
| Sorgono | M89, M170, M26 | 17 | 12 | 12 | 13 | 28 | 23 | 10 | 13 |
| Sorgono | M89, M170, M26 | 15 | 12 | 12 | 14 | 29 | 23 | 10 | 13 |
| Sorgono | M89, M170, M26 | 17 | 11 | 13 | 13 | 28 | 24 | 10 | 13 |
| Sorgono | M89, M170, M26 | 15 | 11 | 12 | 12 | 27 | 23 | 10 | 13 |
| Sorgono | M89, M170, M26 | 17 | 11 | 13 | 13 | 28 | 24 | 10 | 13 |
| Sorgono | M89, M170, M26 | 15 | 12 | 14 | 14 | 29 | 23 | 11 | 14 |
| Sorgono | M89, M172, M102 | 15 | 15 | 15 | 12 | 28 | 24 | 10 | 12 |
| Sorgono | M89, M172, M102 | 15 | 15 | 20 | 13 | 30 | 24 | 10 | 12 |
| Sorgono | M89, M172, M102 | 15 | 15 | 15 | 12 | 28 | 24 | 10 | 12 |
| Sorgono | M89, M172, M67 | 14 | 13 | 16 | 12 | 28 | 23 | 10 | 12 |
| Sorgono | M89, M172, M67 | 15 | 13 | 16 | 12 | 28 | 23 | 10 | 12 |
| Sorgono | M89, M172, M67 | 14 | 13 | 16 | 12 | 28 | 23 | 10 | 12 |
| Sorgono | M89, M201 | 15 | 14 | 14 | 12 | 29 | 23 | 10 | 13 |
| Sorgono | M89, M201 | 15 | 13 | 16 | 12 | 29 | 21 | 10 | 14 |
| Sorgono | M89, M201 | 15 | 11 | 14 | 14 | 30 | 22 | 10 | 13 |
| Sorgono | M89, M201 | 15 | 11 | 15 | 13 | 29 | 22 | 11 | 13 |
| Sorgono | M89, M201 | 15 | 13 | 14 | 12 | 29 | 23 | 11 | 13 |
| Sorgono | M89, M201 | 15 | 14 | 14 | 12 | 29 | 21 | 10 | 14 |
| Sorgono | M89, M201 | 15 | 13 | 15 | 12 | 29 | 23 | 10 | 14 |
| Sorgono | M89, M201 | 15 | 13 | 15 | 11 | 28 | 23 | 10 | 14 |
| Sorgono | M89, M201 | 16 | 13 | 15 | 13 | 30 | 22 | 11 | 14 |
| Sorgono | M89, M201 | 15 | 13 | 14 | 12 | 29 | 23 | 11 | 13 |
| Sorgono | M89, M201 | 15 | 14 | 14 | 12 | 29 | 23 | 10 | 13 |
| Sorgono | M89, M201 | 15 | 11 | 14 | 14 | 30 | 22 | 10 | 13 |
| Sorgono | M89, M267 | 14 | 13 | 20 | 14 | 32 | 23 | 10 | 12 |
| Sorgono | M89, M267 | 14 | 13 | 20 | 13 | 30 | 23 | 10 | 12 |
| Sorgono | M89, M267 | 14 | 13 | 20 | 14 | 32 | 23 | 10 | 12 |
| Sorgono | M89, M9 | 14 | 14 | 18 | 14 | 31 | 23 | 10 | 14 |
| Sorgono | M89, M9 | 14 | 14 | 18 | 14 | 31 | 23 | 10 | 14 |
| Sorgono | M89, M9, M173, M17 | 16 | 11 | 14 | 13 | 30 | 25 | 10 | 13 |
| Sorgono | M89, M9, M173, M17 | 16 | 11 | 14 | 14 | 31 | 24 | 10 | 14 |
| Sorgono | M89, M9, M173, M17 | 16 | 11 | 14 | 14 | 31 | 24 | 10 | 14 |
| Sorgono | M89, M9, M173, M18 | 15 | 11 | 12 | 13 | 28 | 24 | 10 | 13 |
| Sorgono | M89, M9, M173, M18 | 15 | 11 | 12 | 13 | 28 | 24 | 10 | 13 |
| Sorgono | M89, M9, M173, M18 | 15 | 12 | 12 | 13 | 28 | 24 | 10 | 13 |
| Sorgono | M89, M9, M173, M18 | 15 | 12 | 12 | 13 | 28 | 24 | 10 | 13 |
| Sorgono | M89, M9, M173, M18 | 15 | 11 | 12 | 13 | 28 | 24 | 10 | 13 |
| Sorgono | M89, M9, M173, M269 | 15 | 11 | 14 | 13 | 29 | 24 | 10 | 13 |
| Sorgono | M89, M9, M173, M269 | 14 | 11 | 15 | 14 | 30 | 23 | 10 | 13 |
| Sorgono | M89, M9, M173, M269 | 14 | 11 | 14 | 15 | 31 | 23 | 11 | 13 |
| Sorgono | M89, M9, M173, M269 | 14 | 11 | 14 | 14 | 30 | 23 | 11 | 13 |
| Sorgono | M89, M9, M173, M269 | 14 | 11 | 18 | 15 | 31 | 23 | 11 | 13 |
| Sorgono | M89, M9, M173, M269 | 14 | 11 | 16 | 13 | 29 | 24 | 11 | 12 |
| Sorgono | M89, M9, M173, M269 | 14 | 11 | 14 | 13 | 29 | 24 | 11 | 13 |
| Sorgono | M89, M9, M173, M269 | 14 | 11 | 16 | 13 | 29 | 24 | 11 | 12 |
| Sorgono | M89, M9, M173, M269 | 14 | 11 | 14 | 13 | 29 | 24 | 11 | 13 |
| Sorgono | M89, M9, M173, M269 | 14 | 11 | 14 | 14 | 30 | 25 | 11 | 13 |
| Sorgono | M89, M9, M173, M269 | 14 | 11 | 14 | 14 | 30 | 23 | 11 | 13 |
| Sorgono | M89, M9, M173, M269 | 14 | 11 | 14 | 14 | 30 | 23 | 10 | 13 |
| Sorgono | M89, M9, M173, M269 | 14 | 11 | 14 | 14 | 30 | 23 | 11 | 13 |
| Sorgono | M89, M9, M173, M269 | 14 | 11 | 14 | 15 | 31 | 23 | 11 | 13 |
| Sorgono | M89, M9, M173, M269 | 14 | 11 | 15 | 13 | 29 | 24 | 10 | 13 |
| Sorgono | M89, M9, M173, M269 | 14 | 11 | 14 | 13 | 29 | 24 | 11 | 13 |
| Sorgono | M89, M9, M173, M269 | 14 | 11 | 14 | 15 | 31 | 23 | 11 | 13 |
| Sorgono | M89, M9, M173, M269 | 14 | 11 | 14 | 13 | 30 | 24 | 12 | 13 |
| Sorgono | M89, M9, M173, M269 | 14 | 10 | 15 | 13 | 29 | 24 | 10 | 13 |
| Tempio | M1, M35 | 15 | 14 | 16 | 14 | 31 | 23 | 9 | 14 |
| Tempio | M1, M35, M123 | 13 | 16 | 16 | 13 | 30 | 24 | 10 | 12 |
| Tempio | M1, M35, M123 | 13 | 14 | 17 | 13 | 32 | 25 | 10 | 13 |
| Tempio | M1, M35, M123 | 13 | 15 | 17 | 13 | 32 | 25 | 10 | 13 |
| Tempio | M1, M35, M78 | 13 | 18 | 18 | 12 | 29 | 24 | 10 | 13 |
| Tempio | M1, M35, M78 | 13 | 15 | 21 | 13 | 30 | 24 | 10 | 13 |
| Tempio | M1, M35, M78 | 13 | 14 | 17 | 13 | 32 | 25 | 10 | 13 |
| Tempio | M89 | 14 | 12 | 14 | 13 | 31 | 22 | 11 | 15 |
| Tempio | M89 | 14 | 12 | 13 | 13 | 29 | 22 | 10 | 13 |
| Tempio | M89 | 15 | 11 | 14 | 13 | 29 | 23 | 11 | 13 |
| Tempio | M89, M170, M26 | 16 | 12 | 14 | 13 | 28 | 23 | 10 | 13 |
| Tempio | M89, M170, M26 | 17 | 11 | 13 | 14 | 30 | 23 | 10 | 13 |
| Tempio | M89, M170, M26 | 16 | 11 | 13 | 13 | 28 | 24 | 10 | 13 |
| Tempio | M89, M170, M26 | 16 | 13 | 13 | 13 | 28 | 23 | 10 | 13 |
| Tempio | M89, M170, M26 | 17 | 12 | 12 | 13 | 28 | 23 | 10 | 13 |
| Tempio | M89, M170, M26 | 17 | 13 | 13 | 13 | 28 | 23 | 10 | 13 |
| Tempio | M89, M170, M26 | 16 | 12 | 13 | 13 | 28 | 23 | 10 | 13 |
| Tempio | M89, M170, M26 | 17 | 12 | 13 | 13 | 29 | 23 | 10 | 13 |
| Tempio | M89, M170, M26 | 17 | 12 | 12 | 13 | 28 | 21 | 10 | 14 |
| Tempio | M89, M170, M26 | 16 | 12 | 12 | 12 | 28 | 23 | 10 | 13 |
| Tempio | M89, M170, M26 | 16 | 13 | 13 | 13 | 28 | 23 | 10 | 13 |
| Tempio | M89, M170, M26 | 16 | 13 | 13 | 13 | 28 | 23 | 10 | 13 |
| Tempio | M89, M170, M26 | 15 | 12 | 13 | 13 | 28 | 23 | 10 | 13 |
| Tempio | M89, M170, M26 | 17 | 12 | 14 | 13 | 28 | 24 | 10 | 13 |
| Tempio | M89, M170, M26 | 15 | 12 | 13 | 13 | 28 | 23 | 10 | 13 |
| Tempio | M89, M170, M26 | 16 | 12 | 12 | 13 | 28 | 23 | 10 | 13 |
| Tempio | M89, M170, M26 | 17 | 11 | 13 | 13 | 28 | 24 | 10 | 13 |
| Tempio | M89, M170, M26 | 17 | 12 | 12 | 13 | 28 | 23 | 10 | 13 |
| Tempio | M89, M170, M26 | 17 | 12 | 13 | 13 | 28 | 23 | 10 | 14 |
| Tempio | M89, M170, M26 | 16 | 11 | 13 | 13 | 29 | 24 | 10 | 13 |
| Tempio | M89, M170, M26 | 17 | 12 | 13 | 13 | 28 | 24 | 10 | 13 |
| Tempio | M89, M170, M26 | 17 | 12 | 13 | 13 | 28 | 23 | 10 | 13 |
| Tempio | M89, M170, M26 | 15 | 12 | 14 | 13 | 28 | 23 | 10 | 13 |
| Tempio | M89, M172 | 14 | 15 | 17 | 13 | 29 | 23 | 11 | 12 |
| Tempio | M89, M172, M67 | 14 | 13 | 16 | 13 | 31 | 23 | 10 | 12 |
| Tempio | M89, M172, M67 | 14 | 13 | 16 | 13 | 32 | 23 | 10 | 12 |
| Tempio | M89, M172, M67 | 15 | 13 | 17 | 13 | 29 | 23 | 9 | 12 |
| Tempio | M89, M172, M67, M92 | 14 | 12 | 16 | 13 | 29 | 22 | 10 | 12 |
| Tempio | M89, M201 | 15 16 | 13 | 14 | 13 | 31 | 22 | 10 | 14 |
| Tempio | M89, M201 | 15 | 13 | 15 | 12 | 28 | 23 | 10 | 13 |
| Tempio | M89, M201 | 15 16 | 13 | 14 | 13 | 30 | 22 | 10 | 14 |
| Tempio | M89, M201 | 15 | 12 | 14 | 14 | 30 | 22 | 11 | 14 |
| Tempio | M89, M201 | 15 | 14 | 14 | 13 | 30 | 22 | 10 | 13 |
| Tempio | M89, M201 | 15 | 13 | 15 | 12 | 29 | 23 | 10 | 14 |
| Tempio | M89, M201 | 15 | 13 | 15 | 12 | 28 | 23 | 10 | 13 |
| Tempio | M89, M201 | 15 | 12 | 14 | 12 | 29 | 22 | 10 | 14 |
| Tempio | M89, M201 | 15 | 15 | 17 | 12 | 29 | 22 | 10 | 15 |
| Tempio | M89, M201 | 15 | 11 | 12 | 13 | 28 | 22 | 10 | 13 |
| Tempio | M89, M201 | 14 15 | 12 | 14 | 14 | 30 | 22 | 11 | 14 |
| Tempio | M89, M201 | 15 | 13 | 16 | 11 | 28 | 23 | 10 | 14 |
| Tempio | M89, M201 | 15 | 14 | 14 | 13 | 30 | 22 | 10 | 13 |
| Tempio | M89, M9 | 15 | 14 | 16 | 13 | 29 | 23 | 10 | 13 |
| Tempio | M89, M9 | 16 | 13 | 13 | 14 | 29 | 23 | 10 | 13 |
| Tempio | M89, M9 | 14 | 13 | 16 | 13 | 31 | 23 | 10 | 12 |
| Tempio | M89, M9 | 14 | 12 | 16 | 13 | 29 | 22 | 10 | 12 |
| Tempio | M89, M9 | 16 17 | 13 | 14 | 13 | 30 | 22 | 11 | 14 |
| Tempio | M89, M9 | 15 | 14 | 16 | 14 | 31 | 22 | 10 | 13 |
| Tempio | M89, M9, M173, M269 | 14 | 11 | 15 | 14 | 30 | 24 | 11 | 14 |
| Tempio | M89, M9, M173, M269 | 14 | 11 | 15 | 12 | 28 | 24 | 11 | 13 |
| Tempio | M89, M9, M173, M269 | 14 | 11 | 13 | 14 | 30 | 25 | 11 | 13 |
| Tempio | M89, M9, M173, M269 | 14 | 11 | 15 | 13 | 29 | 24 | 11 | 13 |
| Tempio | M89, M9, M173, M269 | 14 | 11 | 13 | 14 | 29 | 24 | 11 | 13 |
| Tempio | M89, M9, M173, M269 | 14 | 12 | 15 | 13 | 29 | 24 | 11 | 13 |
| Tempio | M89, M9, M173, M269 | 14 | 12 | 15 | 13 | 29 | 24 | 11 | 13 |
| Tempio | M89, M9, M173, M269 | 14 | 11 | 14 | 14 | 30 | 24 | 11 | 13 |
| Tempio | M89, M9, M173, M269 | 15 | 11 | 16 | 14 | 30 | 24 | 11 | 13 |
| Tempio | M89, M9, M173, M269 | 15 | 11 | 14 | 14 | 30 | 24 | 11 | 15 |
| Tempio | M89, M9, M173, M269 | 14 | 12 | 14 | 13 | 30 | 24 | 10 | 13 |
| Tempio | M89, M9, M173, M269 | 14 | 11 | 15 | 13 | 29 | 24 | 10 | 13 |
| Tempio | M89, M9, M173, M269 | 14 | 12 | 15 | 13 | 29 | 24 | 11 | 13 |
| Tempio | M89, M9, M173, M269 | 14 | 11 | 14 | 14 | 29 | 24 | 11 | 14 |
| Tempio | M89, M9, M173, M269 | 14 | 12 | 14 | 13 | 30 | 24 | 11 | 13 |
| Tempio | M89, M9, M173, M269 | 14 | 11 | 15 | 13 | 29 | 24 | 11 | 13 |
