# Supplementary material for: Y-Chromosome Based Evidence for Pre-Neolithic Origin of the Genetically Homogeneous but Diverse Sardinian Population: Inference for Association Scans
Source: PLoS One. 2008 Jan 9;3(1):e1430. doi: 10.1371/journal.pone.0001430 (PMC2174525; doi:10.1371/journal.pone.0001430)
Supplement: Table S2 — Conventional Population Pairwise FST values between different European populations (0.06 MB DOC) [file pone.0001430.s002.doc]

**Table S2. Conventional Population Pairwise F_ST_ values between different European populations**

|  | 1 | 2 | 3 | 4 | 5 | 6 | 7 | 8 | 9 | 10 | 11 | 12 | 13 | 14 |
| --- | --- | --- | --- | --- | --- | --- | --- | --- | --- | --- | --- | --- | --- | --- |
| 1 | 0.000 |  |  |  |  |  |  |  |  |  |  |  |  |  |
| 2 | -0.000 | 0.000 |  |  |  |  |  |  |  |  |  |  |  |  |
| 3 | -0.001 | 0.006 | 0.000 |  |  |  |  |  |  |  |  |  |  |  |
| 4 | 0.078 | 0.106 | 0.070 | 0.000 |  |  |  |  |  |  |  |  |  |  |
| 5 | 0.147 | 0.161 | 0.157 | 0.129 | 0.000 |  |  |  |  |  |  |  |  |  |
| 6 | 0.076 | 0.107 | 0.078 | 0.010 | 0.091 | 0.000 |  |  |  |  |  |  |  |  |
| 7 | 0.087 | 0.122 | 0.095 | 0.029 | 0.145 | 0.031 | 0.000 |  |  |  |  |  |  |  |
| 8 | 0.079 | 0.106 | 0.084 | 0.033 | 0.095 | 0.024 | -0.001 | 0.000 |  |  |  |  |  |  |
| 9 | 0.161 | 0.169 | 0.176 | 0.159 | 0.015 | 0.132 | 0.175 | 0.113 | 0.000 |  |  |  |  |  |
| 10 | 0.222 | 0.233 | 0.240 | 0.214 | 0.024 | 0.201 | 0.257 | 0.190 | 0.009 | 0.000 |  |  |  |  |
| 11 | 0.237 | 0.256 | 0.251 | 0.184 | 0.300 | 0.233 | 0.164 | 0.167 | 0.334 | 0.406 | 0.000 |  |  |  |
| 12 | 0.190 | 0.217 | 0.203 | 0.138 | 0.260 | 0.178 | 0.082 | 0.127 | 0.306 | 0.369 | 0.072 | 0.000 |  |  |
| 13 | 0.292 | 0.312 | 0.336 | 0.287 | 0.106 | 0.332 | 0.388 | 0.296 | 0.068 | 0.018 | 0.523 | 0.494 | 0.000 |  |
| 14 | 0.222 | 0.240 | 0.229 | 0.161 | 0.325 | 0.210 | 0.146 | 0.160 | 0.363 | 0.439 | 0.008 | 0.080 | 0.559 | 0.000 |

Label and Population name 1: Cagliari, Southern Sardinia, 2: Sorgono, Central Sardinia; 3: Tempio, Northern Sardinia; 4: Anatolia; 5: Central Italy; 6: Sicily; 7: Albania; 8: Greece; 9: Andalusia; 10: Catalunia; 11: Poland; 12: Croatia; 13: Basque Country; 14: Ukraine (gene frequencies from present work and Semino et al., Science 2000, 290: 1155-9; Bosch et al., Am J Hum Genet 2001, 68: 1019-29; Francalacci et al., Am J Phys Anthropol 2003, 121: 270-9; Cinnioglu et al., Hum Genet 2004, 114: 127-48).
