# Supplementary material for: Y-Chromosome Based Evidence for Pre-Neolithic Origin of the Genetically Homogeneous but Diverse Sardinian Population: Inference for Association Scans
Source: PLoS One. 2008 Jan 9;3(1):e1430. doi: 10.1371/journal.pone.0001430 (PMC2174525; doi:10.1371/journal.pone.0001430)
Supplement: Table S3 — FST P values. Number of permutations: 10100 (0.06 MB DOC) [file pone.0001430.s003.doc]

**Table S3. F_ST_ P values. Number of permutations : 10100**

|  | 1 | 2 | 3 | 4 | 5 | 6 | 7 | 8 | 9 | 10 | 11 | 12 | 13 | 14 |
| --- | --- | --- | --- | --- | --- | --- | --- | --- | --- | --- | --- | --- | --- | --- |
| 1 | * |  |  |  |  |  |  |  |  |  |  |  |  |  |
| 2 | 0.414+-0.005 | * |  |  |  |  |  |  |  |  |  |  |  |  |
| 3 | 0.442+-0.005 | 0.146+-0.003 | * |  |  |  |  |  |  |  |  |  |  |  |
| 4 | 0.000+-0.000 | 0.000+-0.000 | 0.000+-0.000 | * |  |  |  |  |  |  |  |  |  |  |
| 5 | 0.000+-0.000 | 0.000+-0.000 | 0.000+-0.000 | 0.000+-0.000 | * |  |  |  |  |  |  |  |  |  |
| 6 | 0.000+-0.000 | 0.000+-0.000 | 0.000+-0.000 | 0.069+-0.002 | 0.001+-0.000 | * |  |  |  |  |  |  |  |  |
| 7 | 0.000+-0.000 | 0.000+-0.000 | 0.000+-0.000 | 0.002+-0.001 | 0.000+-0.000 | 0.020+-0.001 | * |  |  |  |  |  |  |  |
| 8 | 0.000+-0.000 | 0.000+-0.000 | 0.000+-0.000 | 0.000+-0.000 | 0.000+-0.000 | 0.032+-0.002 | 0.416+-0.005 | * |  |  |  |  |  |  |
| 9 | 0.000+-0.000 | 0.000+-0.000 | 0.000+-0.000 | 0.000+-0.000 | 0.102+-0.003 | 0.000+-0.000 | 0.000+-0.000 | 0.000+-0.000 | * |  |  |  |  |  |
| 10 | 0.000+-0.000 | 0.000+-0.000 | 0.000+-0.000 | 0.000+-0.000 | 0.090+-0.003 | 0.000+-0.000 | 0.000+-0.000 | 0.000+-0.000 | 0.187+-0.004 | * |  |  |  |  |
| 11 | 0.000+-0.000 | 0.000+-0.000 | 0.000+-0.000 | 0.000+-0.000 | 0.000+-0.000 | 0.000+-0.000 | 0.000+-0.000 | 0.000+-0.000 | 0.000+-0.000 | 0.000+-0.000 | * |  |  |  |
| 12 | 0.000+-0.000 | 0.000+-0.000 | 0.000+-0.000 | 0.000+-0.000 | 0.000+-0.000 | 0.000+-0.000 | 0.000+-0.000 | 0.000+-0.000 | 0.000+-0.000 | 0.000+-0.000 | 0.004+-0.000 | * |  |  |
| 13 | 0.000+-0.000 | 0.000+-0.000 | 0.000+-0.000 | 0.000+-0.000 | 0.000+-0.002 | 0.000+-0.000 | 0.000+-0.000 | 0.000+-0.000 | 0.001+-0.000 | 0.109+-0.003 | 0.000+-0.000 | 0.000+-0.000 | * |  |
| 14 | 0.000+-0.000 | 0.000+-0.000 | 0.000+-0.000 | 0.000+-0.000 | 0.000+-0.000 | 0.000+-0.000 | 0.000+-0.000 | 0.000+-0.000 | 0.000+-0.000 | 0.000+-0.000 | 0.219+-0.004 | 0.002+-0.000 | 0.000+-0.000 | * |

Label and Population name 1: Cagliari, Southern Sardinia, 2: Sorgono, Central Sardinia; 3: Tempio, Northern Sardinia; 4: Anatolia; 5: Central Italy; 6: Sicily; 7: Albania; 8: Greece; 9: Andalusia; 10: Catalunia; 11: Poland; 12: Croatia; 13: Basque Country; 14: Ukraine (gene frequencies from present work and Semino et al., Science 2000, 290: 1155-9; Bosch et al., Am J Hum Genet 2001, 68: 1019-29; Francalacci et al., Am J Phys Anthropol 2003, 121: 270-9; Cinnioglu et al., Hum Genet 2004, 114: 127-48).
