# Supplementary material for: Y-Chromosome Based Evidence for Pre-Neolithic Origin of the Genetically Homogeneous but Diverse Sardinian Population: Inference for Association Scans
Source: PLoS One. 2008 Jan 9;3(1):e1430. doi: 10.1371/journal.pone.0001430 (PMC2174525; doi:10.1371/journal.pone.0001430)
Supplement: Table S4 — Pairwise squared size difference RST values between different European populations (0.06 MB DOC) [file pone.0001430.s004.doc]

**Table S4. Pairwise squared size difference** **R_ST_ values between different European populations**

|  | 1 | 2 | 3 | 4 | 5 | 6 | 7 | 8 | 9 | 10 | 11 | 12 | 13 | 14 |
| --- | --- | --- | --- | --- | --- | --- | --- | --- | --- | --- | --- | --- | --- | --- |
| 1 | 0.000 |  |  |  |  |  |  |  |  |  |  |  |  |  |
| 2 | 0.001 | 0.000 |  |  |  |  |  |  |  |  |  |  |  |  |
| 3 | -0.007 | 0.005 | 0.000 |  |  |  |  |  |  |  |  |  |  |  |
| 4 | 0.081 | 0.111 | 0.071 | 0.000 |  |  |  |  |  |  |  |  |  |  |
| 5 | 0.114 | 0.132 | 0.100 | 0.058 | 0.000 |  |  |  |  |  |  |  |  |  |
| 6 | 0.071 | 0.110 | 0.063 | 0.024 | 0.068 | 0.000 |  |  |  |  |  |  |  |  |
| 7 | 0.212 | 0.245 | 0.197 | 0.073 | 0.042 | 0.128 | 0.000 |  |  |  |  |  |  |  |
| 8 | 0.119 | 0.134 | 0.110 | 0.067 | 0.012 | 0.092 | 0.051 | 0.000 |  |  |  |  |  |  |
| 9 | 0.075 | 0.100 | 0.058 | 0.005 | 0.033 | 0.040 | 0.048 | 0.035 | 0.000 |  |  |  |  |  |
| 10 | 0.107 | 0.124 | 0.095 | 0.063 | 0.008 | 0.082 | 0.058 | 0.004 | 0.024 | 0.000 |  |  |  |  |
| 11 | 0.197 | 0.202 | 0.182 | 0.176 | 0.239 | 0.271 | 0.224 | 0.226 | 0.150 | 0.227 |  |  |  |  |
| 12 | 0.159 | 0.184 | 0.138 | 0.095 | 0.170 | 0.184 | 0.151 | 0.172 | 0.069 | 0.158 | 0.034 | 0.000 |  |  |
| 13 | 0.118 | 0.119 | 0.112 | 0.102 | 0.022 | 0.131 | 0.098 | 0.018 | 0.060 | 0.011 | 0.227 | 0.185 | 0.000 |  |
| 14 | 0.238 | 0.251 | 0.223 | 0.200 | 0.280 | 0.306 | 0.279 | 0.292 | 0.175 | 0.278 | -0.001 | 0.036 | 0.291 | 0.000 |

Label and Population name 1: Cagliari, Southern Sardinia; 2: Sorgono, Central Sardinia; 3: Tempio, Northern Sardinia; 4: Anatolia; 5: Tuscany, Central Italy; 6: Sicily, Southern Italy; 7: Albania; 8: Andalusia, Southern Spain; 9: Athens, Greece; 10: Barcelona, Catalonia; 11: Warsaw, Central Poland; 12: Zagreb, Croatia; 13: Pyrenees, Spain; 14: Kiev, Ukraine. Non–Sardinian STR data employed here are from the online reference database of the European Y-chromosomal short tandem repeat (STR) haplotypes (Roewer et al., 2001 Forensic Sci Int 2001, 118:106-13).
