# Supplementary material for: Y-Chromosome Based Evidence for Pre-Neolithic Origin of the Genetically Homogeneous but Diverse Sardinian Population: Inference for Association Scans
Source: PLoS One. 2008 Jan 9;3(1):e1430. doi: 10.1371/journal.pone.0001430 (PMC2174525; doi:10.1371/journal.pone.0001430)
Supplement: Table S5 — RST P values. Number of permutations: 10100 (0.06 MB DOC) [file pone.0001430.s005.doc]

**Table S5. R_ST_ P values. Number of permutations: 10100**

|  | 1 | 2 | 3 | 4 | 5 | 6 | 7 | 8 | 9 | 10 | 11 | 12 | 13 | 14 |
| --- | --- | --- | --- | --- | --- | --- | --- | --- | --- | --- | --- | --- | --- | --- |
| 1 | * |  |  |  |  |  |  |  |  |  |  |  |  |  |
| 2 | 0.310+-0.004 | * |  |  |  |  |  |  |  |  |  |  |  |  |
| 3 | 0.898+-0.003 | 0.217+-0.004 | * |  |  |  |  |  |  |  |  |  |  |  |
| 4 | 0.000+-0.000 | 0.000+-0.000 | 0.000+-0.000 | * |  |  |  |  |  |  |  |  |  |  |
| 5 | 0.000+-0.000 | 0.000+-0.000 | 0.000+-0.000 | 0.000+-0.000 | * |  |  |  |  |  |  |  |  |  |
| 6 | 0.000+-0.000 | 0.000+-0.000 | 0.000+-0.000 | 0.000+-0.000 | 0.000+-0.000 | * |  |  |  |  |  |  |  |  |
| 7 | 0.000+-0.000 | 0.000+-0.000 | 0.000+-0.000 | 0.000+-0.000 | 0.000+-0.000 | 0.000+-0.000 | * |  |  |  |  |  |  |  |
| 8 | 0.000+-0.000 | 0.000+-0.000 | 0.000+-0.000 | 0.000+-0.000 | 0.005+-0.000 | 0.000+-0.000 | 0.000+-0.000 | * |  |  |  |  |  |  |
| 9 | 0.000+-0.000 | 0.000+-0.000 | 0.000+-0.000 | 0.165+-0.004 | 0.000+-0.000 | 0.000+-0.000 | 0.000+-0.000 | 0.000+-0.000 | * |  |  |  |  |  |
| 10 | 0.000+-0.000 | 0.000+-0.000 | 0.000+-0.000 | 0.000+-0.000 | 0.018+-0.001 | 0.000+-0.000 | 0.000+-0.000 | 0.104+-0.003 | 0.000+-0.000 | * |  |  |  |  |
| 11 | 0.000+-0.000 | 0.000+-0.000 | 0.000+-0.000 | 0.000+-0.000 | 0.000+-0.000 | 0.000+-0.000 | 0.000+-0.000 | 0.000+-0.000 | 0.000+-0.000 | 0.000+-0.000 | * |  |  |  |
| 12 | 0.000+-0.000 | 0.000+-0.000 | 0.000+-0.000 | 0.000+-0.000 | 0.000+-0.000 | 0.000+-0.000 | 0.000+-0.000 | 0.000+-0.000 | 0.000+-0.000 | 0.000+-0.000 | 0.000+-0.000 | * |  |  |
| 13 | 0.000+-0.000 | 0.000+-0.000 | 0.000+-0.000 | 0.000+-0.000 | 0.001+-0.000 | 0.000+-0.000 | 0.000+-0.000 | 0.002+-0.000 | 0.000+-0.000 | 0.013+-0.001 | 0.000+-0.000 | 0.000+-0.000 | * |  |
| 14 | 0.000+-0.000 | 0.000+-0.000 | 0.000+-0.000 | 0.000+-0.000 | 0.000+-0.000 | 0.000+-0.000 | 0.000+-0.000 | 0.000+-0.000 | 0.000+-0.000 | 0.000+-0.000 | 0.453+-0.005 | 0.001+-0.000 | 0.000+-0.000 | * |

Label and Population name 1: Cagliari, Southern Sardinia; 2: Sorgono, Central Sardinia; 3: Tempio, Northern Sardinia; 4: Anatolia; 5: Tuscany, Central Italy; 6: Sicily, Southern Italy; 7: Albania; 8: Andalusia, Southern Spain; 9: Athens, Greece; 10: Barcelona, Catalonia; 11: Warsaw, Central Poland; 12: Zagreb, Croatia; 13: Pyrenees, Spain; 14: Kiev, Ukraine. Non–Sardinian STR data employed here are from the online reference database of the European Y-chromosomal short tandem repeat (STR) haplotypes (Roewer et al., 2001 Forensic Sci Int 2001, 118:106-13).
