# Supplementary material for: Y-Chromosome Based Evidence for Pre-Neolithic Origin of the Genetically Homogeneous but Diverse Sardinian Population: Inference for Association Scans
Source: PLoS One. 2008 Jan 9;3(1):e1430. doi: 10.1371/journal.pone.0001430 (PMC2174525; doi:10.1371/journal.pone.0001430)
Supplement: Table S6 — AMOVA analysis of 12 pre-molecular markers on 21 linguistic domains of Sardinia (0.05 MB DOC) [file pone.0001430.s006.doc]

**Table S6. AMOVA analysis of 12 pre-molecular markers on 21 linguistic domains of Sardinia**

| Marker | Fixation Index F_ST_ | P (rand. value > = obs. value) | Percentage of variation | Among populations | Within populations |
| --- | --- | --- | --- | --- | --- |
| AB0 | 0.000 | 0.575+-0.006 |  | -0.04 | 100.04 |
| ACP1 | 0.002 | 0.102+-0.003 |  | 0.15 | 99.85 |
| AK1 | 0.003 | 0.062+-0.002 |  | 0.15 | 99.85 |
| C3 | 0.002 | 0.140+-0.003 |  | 0.24 | 99.76 |
| CDE | 0.006 | 0.000+-0.000 |  | 0.56 | 99.44 |
| DIA | 0.002 | 0.096+-0.003 |  | 0.23 | 99.77 |
| ESD | 0.004 | 0.014+-0.001 |  | 0.36 | 99.64 |
| GC | 0.001 | 0.311+-0.005 |  | 0.08 | 99.92 |
| KELL | -0.002 | 0.780+-0.005 |  | -0.19 | 100.19 |
| MN | 0.002 | 0.206+-0.004 |  | 0.18 | 99.82 |
| PGM1 | 0.001 | 0.217+-0.004 |  | 0.11 | 99.89 |
| 6PGD | 0.001 | 0.314+-0.005 |  | 0.06 | 99.94 |
| Average over genetic markers | 0.002 |  |  | 0.16 | 99.84 |

Values are re-computed using the Arlequin software from the allele frequencies reported in Table 1 on page 130 of Cappello et al. Ann Hum Genet 1996; 60:125-41.
